# Antimicrobial activity and nanoremediation of heavy metals using biosynthesized CS/GO/ZnO nanocomposite by *Bacillus subtilis* ATCC 6633 alone or immobilized in a macroporous cryogel

**DOI:** 10.1186/s12934-024-02535-6

**Published:** 2024-10-15

**Authors:** Mohamed M. El-Zahed, Mohamed I. Abou-Dobara, Marwa M. El-Khodary, Mohamed M. A. Mousa

**Affiliations:** Department of Botany and Microbiology, Faculty of Science, Damietta University, New Damietta, 34517 Egypt

**Keywords:** Zinc oxide, Graphene oxide, Chitosan, Nanocomposite, *Bacillus subtilis*, Antimicrobial, Cryogel, Water treatment

## Abstract

**Background:**

The world society is still suffering greatly from waterborne infections, with developing countries bearing most of the morbidity and death burden, especially concerning young children. Moreover, microbial resistance is one of the most prevalent global problems that extends the need for self-medication and the healing period, or it may be linked to treatment failure that results in further hospitalization, higher healthcare expenses, and higher mortality rates. Thus, innovative synthesis of new antimicrobial materials is required to preserve the environment and enhance human health.

**Results:**

The present study highlighted a simple and cost-effective approach to biosynthesize a chitosan/graphene oxide/zinc oxide nanocomposite (CS/GO/ZnO) alone and immobilized in a macroporous cryogel as a new antimicrobial agent. *Bacillus subtilis* ATCC 6633 was used as a safe and efficient bio-nano-factory during biosynthesis. The formation of CS/GO/ZnO was confirmed and characterized using different analyses including ultraviolet-visible spectroscopy (UV-Vis), X-ray diffraction (XRD), Fourier transform infrared spectroscopy (FTIR), selective area diffraction pattern (SADP), Zeta analyses, scanning electron microscope (SEM) and transmission electron microscopy (TEM). GO combined with ZnO NPs successfully and displayed an adsorption peak at 358 nm. The XRD results showed the crystalline composition of the loaded ZnO NPs on GO sheets. FTIR spectrum confirmed the presence of proteins during the synthesis which act as stabilizing and capping agents. The nanocomposite has a high negative surface charge (-32.8 ± 5.7 mV) which increases its stability. SEM and TEM showing the size of biosynthesized ZnO-NPs was in the range of 40–50 nm. The CS/GO/ZnO alone or immobilized in cryogel revealed good antimicrobial activities against *B. cereus* ATCC 14,579, *Escherichia coli* ATCC 25,922, and *Candida albicans* ATCC 10,231 in a dose-dependent manner. The CS/GO/ZnO cryogel revealed higher antimicrobial activity than GO/ZnO nanocomposite and standard antibiotics (amoxicillin and miconazole) with inhibition zones averages of 24.33 ± 0.12, 15.67 ± 0.03, and 17.5 ± 0.49 mm, respectively. The MIC values of the prepared nanocomposite against *B. cereus*, *E. coli*, and *C. albicans* were 80, 80, and 90 µg/ml compared to standard drugs (90, 120 and 150 µg/ml, respectively). According to the TEM ultrastructure studies of nanocomposite-treated microbes, treated cells had severe deformities and morphological alterations compared to the untreated cells including cell wall distortion, the separation between the cell wall and plasma membrane, vacuoles formation moreover complete cell lyses were also noted. In the cytotoxicity test of CS/GO/ZnO alone and its cryogel, there was a significant reduction (*p*˂0.05) in cell viability of WI-38 normal lung cell line after the concentration of 209 and 164 µg/ml, respectively. It showed the low toxic effect of the nanocomposite and its cryogel on the WI-38 line which implies its safety. In addition, water treatment with the CS/GO/ZnO cryogel decreased turbidity (0.58 NTU), total coliform (2 CFU/100 ml), fecal coliform (1 CFU/100 ml), fecal *Streptococcus* (2 CFU/100 ml), and heterotrophic plate counts (53 CFU/1 ml) not only in comparison with the chlorine-treated samples (1.69 NTU, 4 CFU/100 ml, 6 CFU/100 ml, 57 CFU/100 ml, and 140 CFU/1 ml, respectively) but also with the raw water samples (6.9 NTU, 10800 CFU/100 ml, 660 CFU/100 ml, 800 CFU/100 ml, and 4400 CFU/1 ml, respectively). Moreover, cryogel significantly decreased the concentration of different heavy metals, especially cobalt compared to chlorine (0.004 ppm, 0.002 ppm, and 0.001 ppm for raw water, chlorine-treated, and cryogel-treated groups, respectively) which helped in the reduction of their toxic effects.

**Conclusion:**

This study provides an effective, promising, safe, and alternative nanocomposite to treat different human and animal pathogenic microbes that might be used in different environmental, industrial, and medical applications.

## Background

Water quality and quantity are major issues facing the world today. Nowadays, there is an increase in waterborne diseases due to the poor water quality. One of the main reasons for the rise in the death rate is water-related disorders brought on by pathogenic microbes [1]. These microbes are transmitted to humans through recreational activities and contaminated water. Ensuring the public’s health requires appropriate and sufficient water treatment to lower the likelihood of waterborne diseases [2]. According to research published by the WHO in June 2019, at least 2 billion people suffer from utilizing drinking water sources contaminated with hazardous chemicals and feces [3]. Diseases including typhoid, cholera, polio, and dysentery can be spread via contaminated water. Also, 485,000 deaths from diarrhea are thought to occur annually as a result of contaminated drinking water. The WHO further projects that half of the world’s population will reside in water-stressed areas by 2025. These data highlight the serious health risks and diseases that come with water scarcity, which includes both the availability and quality of water for the world’s population.

Enhancements in drinking water, treatment, sanitation, hygiene, and water resource management have the potential to lower the worldwide disease burden by 10% [4]. Thus, the goal of any water treatment procedure is to rid the water of impurities including microbes and hazardous chemicals to prepare it for its intended purpose. Chemical, physical, and biological techniques are improved and applied in water treatment to get rid of chemical and biological impurities. Disinfection is a critical phase in the water treatment procedure that eliminates inactive waterborne microbes [5]. The two types of disinfection that are most frequently employed in water treatment are chemical disinfection (such as chlorine dioxide, ozone, and chloramine) and physical disinfection (such as UV radiation) [6]. Pools, drinking water, and wastewater have all traditionally been treated with disinfectants. Because it eliminates dangerous bacteria, it is therefore essential to the drinking water treatment process and safeguards public health [7]. Nevertheless, disinfectants were reported to have significant worries due to their high cost, toxicity, dependability, efficacy, and generation of toxic by-products [8]. On the other hand, conventional treatment techniques such as membrane filtration, biological approach, sedimentation, and coagulation are comparatively unsuccessful due to the high toxicity of water-soluble contaminants [8–10]. Alternative disinfection techniques that can accomplish effective results while being portable, broad-spectrum, environmentally benign, energy-efficient, easy to use, and commercially feasible are still desperately needed.

Hydrogels and cryogels have been studied and introduced as alternative treatment techniques for water and wastewater treatment owing to their recyclability, low cost, and ease of preparation [11]. A three-dimensional (3D) porous cross-linked network with solid-like properties is referred to as a “hydrogel” because it can retain a significant amount of water or biological fluids while retaining its structural and functional integrity in a variety of environmental conditions or when exposed to external stresses, such as light, temperature, mechanical forces, electric or magnetic fields, and so on [11, 12]. They can be broadly categorized into three classes: hydrogel films, hydrogel beads, and hydrogel nanocomposites. However, the use of toxic materials, poor degradation, brittleness, inadequate gel integrity, rigidity, long-time removal consuming technique, and occluded flow homogeneity diffusion for chemical and microbes coupling have been major concerns limiting their applications [11–13]. In contrast to hydrogels, cryogels readily have a highly interconnected 3D macroporous structure. Cryogelation is one of the simplest, cheapest, easiest, and fastest techniques enabling the fabrication of biodegradable and safe materials with unique characteristics [13]. At below-freezing temperatures, cryogels are crosslinked, causing the solvent crystals to function as porogens. Because of their porous structure, cryogel can retain debris, toxins, and pollutants from contaminated water [13–15]. Cryogels and hydrogels with antimicrobial properties can be loaded with biological extracts, antibiotics, metal and metal oxide nanoparticles (NPs), or antimicrobial peptides [16]. Antimicrobial cryogels offer a three-dimensional (3D) framework that promotes sustained drug release, allowing for the localized and regulated delivery of antimicrobial compounds due to their gel-like structure [17]. Metallic NPs stand out among these antimicrobial materials because of their unique characteristics like a high surface area to volume ratio, high selectivity, and efficacy [18, 19].

Unlike traditional water disinfection technology, nanomaterials can be created as point-of-use water inert in a water environment and reduce the possibility of producing hazardous by-products [20]. Since their surface area is increased, metal materials at the nanoscale can exhibit a range of enhanced properties compared to their bulk scale (1 to 100 nm) [21, 22]. Because of their antibacterial qualities, zinc oxide nanoparticles (ZnO NPs) are one type of metal oxide NPs that has drawn a lot of interest. ZnO NPs have several beneficial characteristics, including chemical stability, low cost, a large surface area, ease of preparation, selectivity, heat resistance, and potent antimicrobial activity against different pathogenic microbial strains, thermoresistant spores, and human cells without causing toxicity [23–27]. To prevent microbial development, ZnO NPs are also used as antimicrobials for cellulose fibers, water treatment, textiles, surface coatings, and cosmetics [28–31]. Under this concept, a wealth of literature exists describing ZnO NPs’ antibacterial efficacy against a variety of pathogens, such as *Candida albicans*, *Pseudomonas aeruginosa*, *Escherichia coli*, *Enterococcus faecalis*, *Klebsiella pneumoniae*, and *Staphylococcus aureus* [32–35]. ZnO NPs can be produced using a variety of physical, chemical, or biological techniques [36, 37]. Biological methods, on the other hand, are more widely accepted than other approaches since they are simpler, less expensive, biocompatible, and environmentally benign because they don’t utilize any toxic materials [38]. These methods can be accomplished with the aid of plants, bacteria, fungi, or their enzymes. Bacterial synthesis is a more advantageous biological process than the others due to its easy manipulation of cells and faster development rate [39]. Therefore, it is thought of bacteria as characteristic nano-factories. One of two ways that bacteria can synthesize NPs is extracellularly or intracellularly. Because it does not require downstream processing, the extracellular approach is favorable moreover its simplicity and low cost [40].

Stability and aggregation are two frequent issues that reduce the utilization of nanometals in water treatment applications. Embedding in polymers and forming a corporation are two recommendations [41, 42]. Graphene oxide (GO) is a low-toxicity carbon polymer. Recent research has indicated that because graphene and graphene-based nanocomposites have little to no cytotoxicity to human and animal cells, they could be employed as antibacterial coatings for food goods and water treatment [43]. For fiber-reinforced polymer composites, GO has been widely employed as a nanofiller [44]. Particular uses of GO included the elimination of metal ions, turbidity, and salts from water [45–47]. It has been observed that GO nanomaterials exhibit bactericidal action against a wide range of Gram-negative and Gram-positive bacteria [48, 49].

According to the super individual properties of GO and ZnO, combining them can enhance their performances as antimicrobial agents as well as their capacity to treat water. Thus, the current study aimed to synthesize CS/GO/ZnO immobilized in macroporous cryogel and test its antimicrobial action as well as heavy metal nanoremediation in water treatment.

## Materials and methods

### Materials

Zinc nitrate hexahydrate salt (99.9%) and chitosan (w/v, MW 50–190 KDa, deacetylation degree: ≥85%) were provided from Techno Pharmchem., India. Graphite fine powder (98%, 230 mesh size) was purchased from LOBA Chemie. Pvt. Ltd, India. All chemicals and solvents were of analytical grade.

### Microbial strains

Culture slants of *Bacillus subtilis* ATCC 6633, *B. cereus* ATCC 14,579, *Escherichia coli* ATCC 25,922, and *Candida albicans* ATCC 10,231 were obtained from the Microbiology Laboratory, Faculty of Science, Damietta University (Damietta, Egypt) and sub-cultured on nutrient agar plates for 24 h at 37 °C before use.

### Methods

#### Extracellular biosynthesis of ZnO NPs

A significantly modified protocol based on Hamk et al. [50] study was used to synthesize ZnO NPs employing cell-free bacterial supernatant of *B. subtilis* ATCC 6633 as a safe and quick bio-nano-factory. The chosen strain was grown to a 0.5 McFarland standard (1–2 × 10^8^ CFU/ml) and then inoculated into 250 ml conical flasks with 100 ml of autoclaved nutrient broth medium. The flasks were incubated for 24 h at 37 °C and 150 rpm. Bacterial supernatants were collected by centrifuging at 5000 rpm for 20 min and filtering through a 0.2-µm syringe filter. A 6 mM stock solution of zinc nitrate was prepared in distilled water and mixed with the cell-free supernatant of *B. subtilis* ATCC 6633. At room temperature, a few drops from NaOH solution (5 mM) were added to maintain the pH of this reaction mixture between 9 and 10. The reaction mixture was incubated at 150 rpm and 37 °C for the entire night. When ZnO NPs develop, the apparent color changes from colorless to yellowish-white. Centrifugation was used for 20 min at 5000 rpm to collect the yellowish-white precipitate of ZnO NPs. After repeatedly washing the resulting NPs in distilled water to get rid of any undesired materials, they were oven-dried for 8 h at 80 °C. Before being characterized, the synthesized NPs were calcined for 3 h at 550 °C in a muffle furnace.

### Synthesis of graphene oxide (GO) sheets

Graphite powder (Gt) was oxidized using strong oxidizing chemicals as part of the GO production technique. Here, the pristine Gt was oxidized in accordance with the Hummers and Offeman method [51] to produce the GO. In an ice-water bath, 50 ml of concentrated sulfuric acid and 2 g of Gt were mixed and stirred for 2 h at 35 °C. Next, 16.6 ml of concentrated nitric acid was added, the mixture was heated for 3 min to 80 °C, and the mixture was stirred for 1.5 h. The mixture was diluted with 350 ml of distilled water. Drop by drop, 20 ml of 30% hydrogen peroxide was added to the reaction mixture. To get the final GO to have a pH of 7, it was first washed with a 5% HCl solution and then with distilled water. The mixture underwent a 15-min centrifugation at 10,000 rpm and a 24-h drying process at 60 °C in an oven.

### Decoration of ZnO NPs on GO sheets (GO/ZnO)

Different amounts of ZnO NPs (0.005, 0.015, and 0.025 g) were decorated on the GO sheets (0.5 g) with loading percentages of 1%, 3%, and 5%. The decoration process was fabricated using a simple sol-gel method in a gelatine medium, which was used as a polymerization agent [52]. Firstly, 1.25 g of gelatine was added to 50 ml of distilled water at 60ºC to obtain a gelatine solution. In another beaker, GO was dispersed and stirred in a minimum cell-free bacterial supernatant of *B. subtilis* ATCC 6633 at room temperature. ZnO NPs were added into the GO solution under stirring at 200 rpm and room temperature. Lastly, the prior combination was mixed with the gelatine solution. The mixture was heated to 80ºC while stirred at 200 rpm, resulting in the formation of a gelatinous phase that was dark brown in color. After drying, the mixture was calcined for 1 h at 500ºC.

### Preparation of CS/GO/ZnO cryogel

Firstly, to prepare CS/GO/ZnO hydrogel, a described earlier method by Agnihotri et al. [53] was used with some modifications. 0.25 g chitosan (CS) was dissolved in 12.5 ml acetic acid (1%) at 80 °C until complete dissolution. 40, 80, and 120 mg of GO/ZnO were added separately to 20 ml cell-free bacterial supernatant of *B. subtilis* ATCC 6633 and stirred for 15 min at room temperature. The nanocomposite solutions were added separately to the CS solution and stirred for complete homogeneity. 1.5 g of polyvinyl alcohol (PVA) was dissolved in 16 ml of distilled water at 80 °C and stirred for 15 min until the solution became clear and homogeneous. Thereafter, nanocomposite solutions were added to the PVA solution and stirred for 15 min at 40 °C. Finally, 1 ml glutaraldehyde was added to the reaction solutions and then poured into a mold. To prepare the CS/GO/ZnO cryogels, five consecutive cycles of freezing and thawing were applied to the hydrogels to create porous network structures within the system and improve the chain entanglement of the hydrogel’s polymeric species [54]. Each cycle consisted of a 16-h freezing phase at -20 °C followed by a 3-h thawing step at 25 °C.

### Characterization

The formation of ZnO, GO and GO/ZnO was studied using ultraviolet-visible (UV-vis) (UV/VIS/NIR Spectrophotometer V-630, Japan). Fourier transform-infrared (FTIR) (FT/IR-4100typeA) spectra were used to analyze ZnO, GO, and GO/ZnO formation. The morphological and crystallographic investigations were performed using transmission electron microscopy (TEM, JEOL JEM-2100, Japan). Zeta potential analysis (Malvern Instruments Ltd; zs90, Worcestershire, UK) was done to measure the potential of GO/ZnO. X-ray diffraction patterns (XRD) (model LabX XRD-6000, Shimadzu, Japan) of ZnO, GO, and GO/ZnO formation were also studied. SEM observations were evaluated to study the physical properties of GO/ZnO in the PVA matrix.

### Evaluation of the antimicrobial activity of GO/ZnO nanocomposite and cryogel

The antimicrobial actions of GO/ZnO nanocomposite were tested against different microbial models including Gram-positive bacterium *B. cereus* ATCC 14,579, Gram-negative bacterium *E. coli* ATCC 25,922, and a pathogenic yeast *C. albicans* ATCC 10,231 using the agar well diffusion method [55]. Using the spread plate technique, Mueller Hinton agar (MHA) plates were made and inoculated with a 0.5 McFarland standard derived from tested microorganisms. 100 µl of GO/ZnO solutions (1%, 3%, and 5%) were prepared and added into wells (5 mm) in the MHA plates. The agar plates were incubated at 37 °C for 24 h. After the incubation period, zones of inhibition (ZOI) were calculated in mm.

Similarly, the antibacterial activity of CS/GO/ZnO cryogels was tested in a solid medium through the disc diffusion method [56]. MHA agar plates were spread plated with 0.5 McFarland standard of tested microbial strain. 5 mm discs of CS/GO/ZnO cryogels were prepared, placed on the surface of inoculated agar plates, and incubated at 37˚C for 24 h. ZOI was calculated in mm after the incubation. Standard antibiotics amoxicillin (AMX) and miconazole discs (120 mg) were also used as positive controls.

### Minimum inhibition concentration (MIC)

The MIC for the synthesized GO/ZnO nanocomposite was studied using the broth dilution method [57]. Using a 0.5 McFarland standard of the investigated microbial strain, nutrient broth media flasks enriched with a range of GO/ZnO concentrations (0–150 µg/ml) were inoculated. The inoculated flasks were incubated aerobically at 37 °C and 150 rpm for 24 h. The negative (no nanocomposite) and positive (AMX and miconazole) controls were also tested. The MIC values of GO/ZnO were recorded with no apparent microbial growth. MIC values were confirmed spectrophotometrically at 600 nm against blank.

### In vitro cytotoxicity test of CS/GO/ZnO cryogel

The cytotoxic effect of CS/GO/ZnO nanocomposite and its cryogel was tested using a human fibroblast WI-38 cell line which purchased from the American Type Culture Collection (ATCC, Rockville, MD). The cells were cultivated in a Dulbecco’s modified Eagle’s medium (DMEM, Lonza, USA) containing 10% fetal bovine serum, 50 µg/ml gentamycin, 1% L-glutamine, and HEPES buffer at the Regional Centre for Mycology and Biotechnology (Al-Azhar University, Cairo). The cells were kept in a humidified incubator with 5% CO_2_ at 37 °C. The cytotoxic effect of CS/GO/ZnO cryogel was investigated using 3-(4,5-dimethyl thiazol-2-yl)-2,5-diphenyl tetrazolium bromide (MTT) cell viability assay and the optical density (O.D) was measured using a microplate reader (SunRise, TECAN, Inc, USA) at 590 nm. The cell viability was calculated from the equation: Cytotoxicity (%) = [(O.D of control cells − O.D of treated cells)/O.D of control cells)] × 100. The CC_50_ values were calculated using a linear equation from a plot between the percentages of cell viability vs. concentrations [58].

### Water treatment and heavy metals nanoremediation

At the start of 2024, raw water samples were collected under aseptic conditions from the Nile River, Damietta branch, Damietta Government, Egypt (31°23’59.4"N 31°46’35.6” E) and immediately transferred to the Microbiology laboratory, Botany and Microbiology Department, Faculty of Science, Damietta University. The water temperature, pH, conductivity, turbidity, total dissolved solids (TDS), residual aluminum concentration, and initial metal concentrations such as copper, manganese, zinc, iron, and cobalt were measured were investigated. The total coliform (CFU/100 ml), fecal coliform (CFU/100 ml), fecal *Streptococcus* (CFU/100 ml), and heterotrophic plate count (CFU/1 ml) of water samples were also measured. Water samples were divided into 3 groups; the 1st group was treated with 5 mm discs of CS/GO/ZnO cryogels (30 discs/500 ml, supplemented by 80 mg of GO/ZnO), the 2nd group was treated with chlorine (6 ppm) and ammonium alum (22 mg/l), while the 3rd was assigned to the control group without treatment. Water samples were exposed to their specific treatments for 15 min at room temperature (25 °C) and 90 rpm. Finally, the physical, chemical, and biological measures were also determined after the treatment and compared to the recommended limits by WHO and APHA standards [59, 60].

### Statistical analysis

Statistical analysis was performed on the data using SPSS version 18 software. Every experiment’s value was reported as the mean ± standard deviation (SD), and one-way analysis of variance (ANOVA) was used for analysis followed by Duncan’s multiple range test. A significant threshold of *p* < 0.05 was applied [61]. All experiments were performed at least 3 times and done in triplicate.

## Results

### Synthesis and characterization of ZnO NPs, GO, GO/ZnO nanocomposite and cryogel

**Fig. 1:**
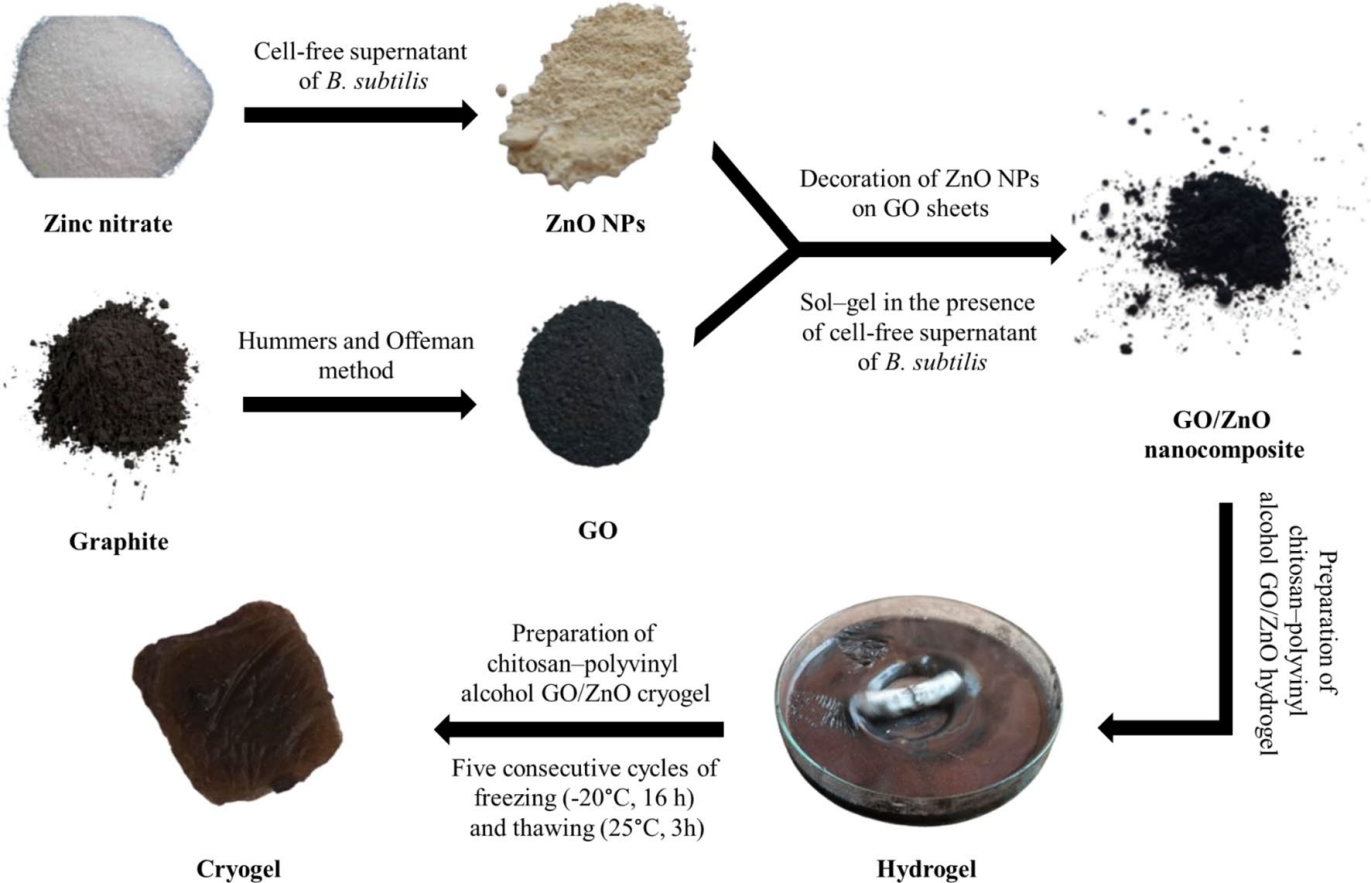
Schematic of ZnO NPs, GO, GO/ZnO nanocomposite, hydrogel, and cryogel preparation processes

**Fig. 2:**
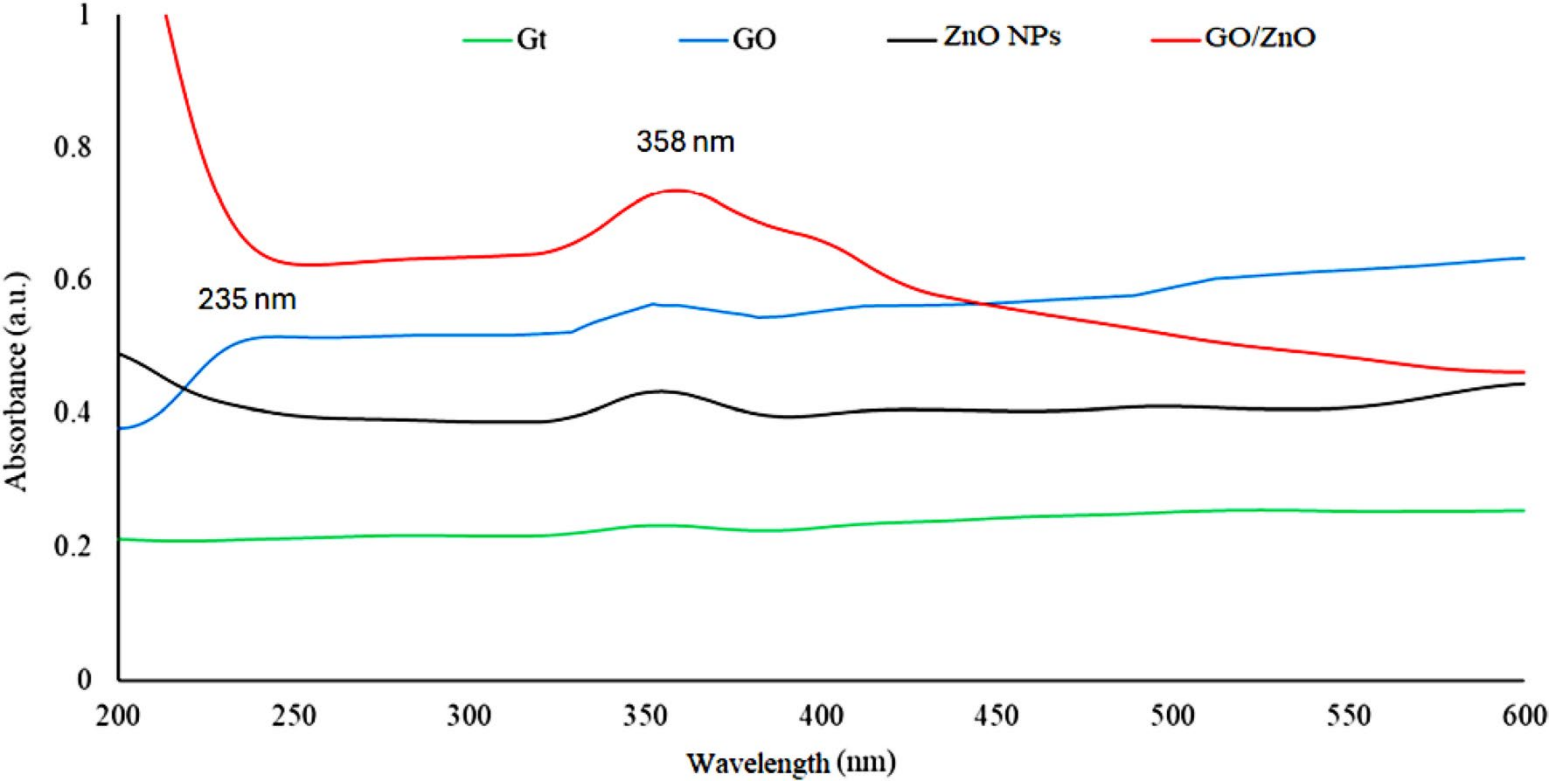
The UV-Vis spectra of Gt, GO, ZnO NPs, and GO/ZnO nanocomposite

As a first observation for NPs biosynthesis, the color changes from colorless to yellowish-white, confirming the formation of ZnO NPs (Fig. 1). At 37 °C and 150 rpm, *B. subtilis* could biosynthesize ZnO NPs in less than 24 h, resulting in a large absorption peak in the 350–368 nm range, which is a distinguishing band for pure ZnO NPs [46]. While the adsorption peak at 358 nm confirmed the successful combination between ZnO NPs and GO sheets (Fig. 2) [52].

**Fig. 3:**
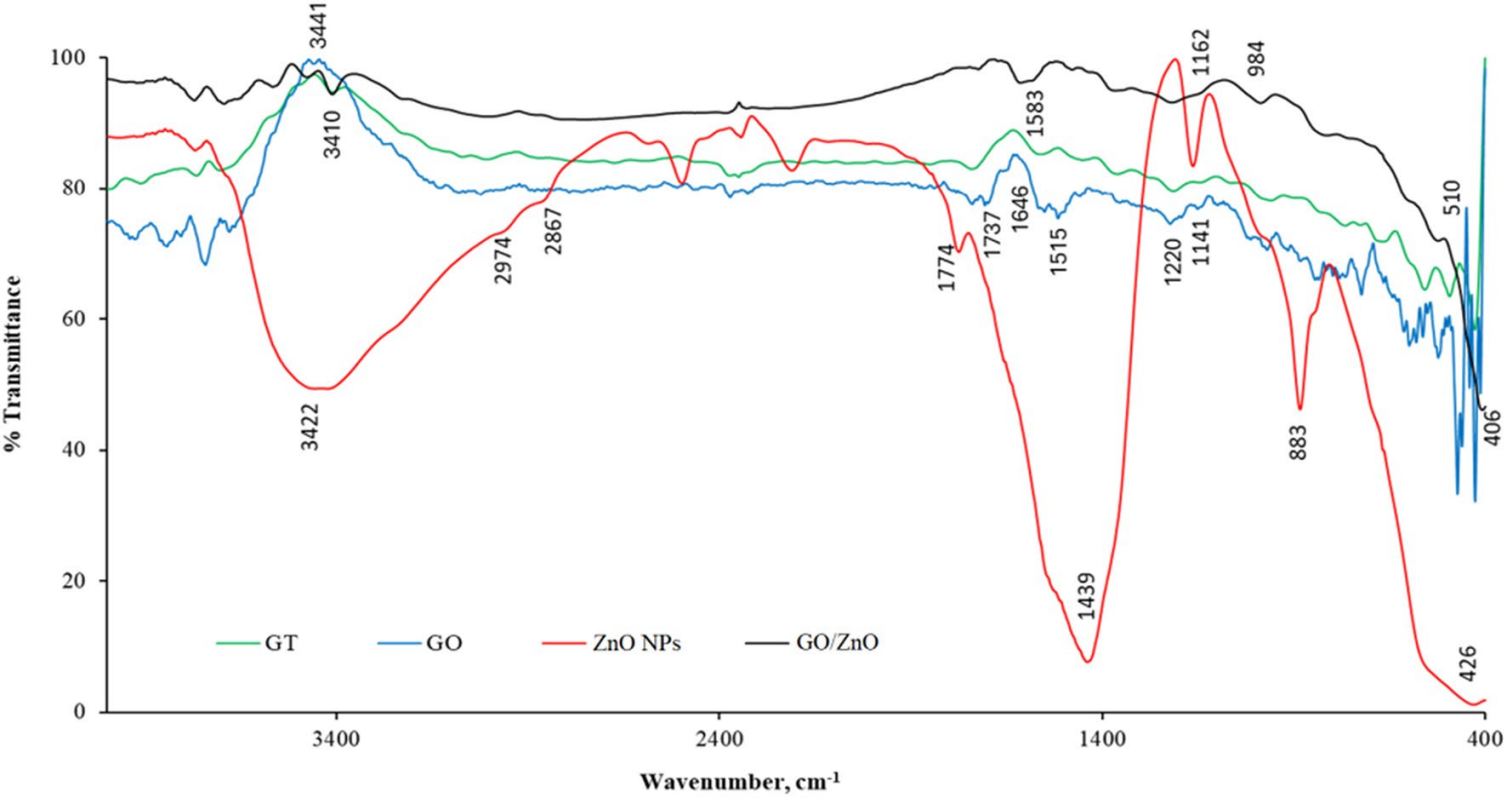
The FTIR spectra of Gt, GO, ZnO NPs, and GO/ZnO nanocomposite

FTIR spectra of ZnO NPs and GO/ZnO nanocomposite were measured and confirmed the presence of proteins during their fabrication at 2867, 2974,1774, 1439, 1162, 883 cm^-1^ for the bond of NH_2_, adenine, cytosine, and guanine (Fig. 3). Stretching vibrations of metal-oxygen appeared between 400 and 600 cm^-1^ (428 cm^-1^) indicating the presence of ZnO NPs [25]. The OH groups appeared at ≈ 3422 cm^-1^. Gt was oxidized into GO as shown in the FTIR spectra (Fig. 3). The characteristic peaks of hydroxyl (OH stretching), carboxyl (C = O stretching), Alkenes (C = C stretching) and carbonyl (= C = O stretching) appeared at 3441, 1737, 1631, 1515, 1220,1141, 1054 cm^− 1^, respectively. Functional groups that include oxygen, such as hydroxyl, carboxyl, and carbonyl, demonstrated that Gt powder was successfully oxidized to GO. The decrease in the intensity of vibration bands of − OH, NH_2_, and C–N groups at GO/ZnO nanocomposite FTIR spectrum indicated their involvement in the bonding with ZnO NPs.

**Fig. 4:**
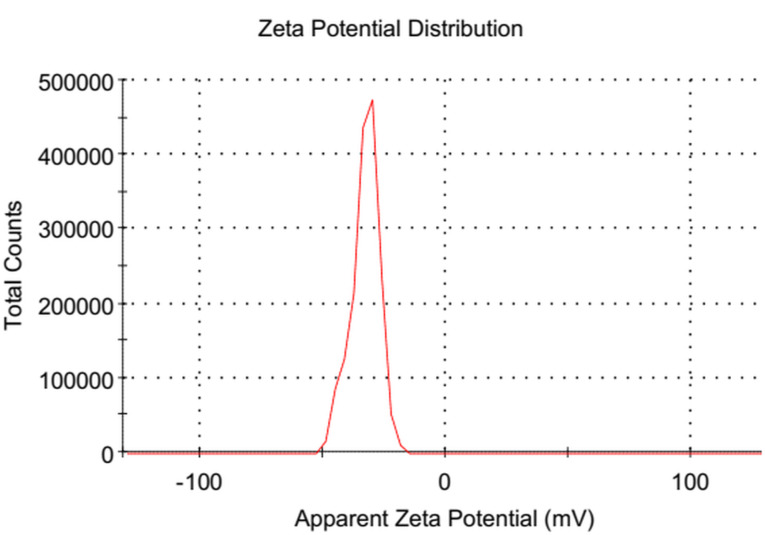
The Zeta potential analysis of GO/ZnO nanocomposite

The results of Zeta potential analysis displayed the high negative charge of the prepared nanocomposite which reached − 32.8 ± 5.7 mV (Fig. 4).

**Fig. 5:**
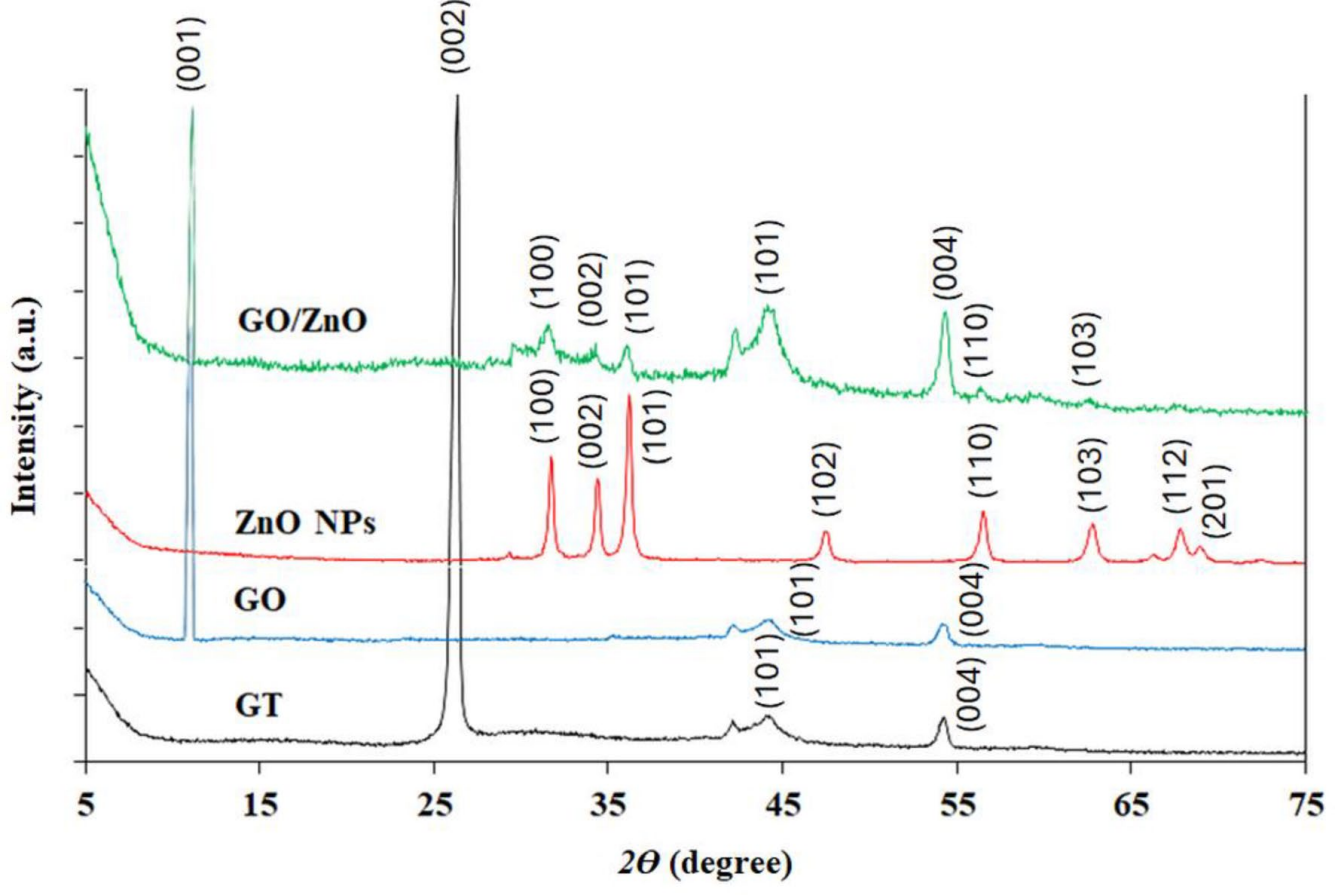
XRD of Gt, GO, ZnO NPs, and GO/ZnO nanocomposite

The XRD of Gt, GO, ZnO NPs, and GO/ZnO nanocomposite were analyzed between 10° and 80° in the 2*θ* range (Fig. 5). The XRD pattern of Gt was indexed with the support of JCPDS card No. 41–1487. The results indicated the presence of a strong peak at 2*θ* = 26.35° which was specific to the (002) plane with *d*-spacing of 3.38 Å and signifies that Gt is a highly oriented carbon material. Also, peaks at 2*θ* = 44.17° (*d*-spacing of 2.05 Å) and 54.19° (*d*-spacing of 1.69 Å) were observed that were specific to the (101) and (004) planes and indicate the crystalline structure of graphite. The (001) characteristic peak at 2*θ* = 11.7° (*d*-spacing of 8.8 Å) was visible in the XRD pattern of GO, confirming the proximity of oxygen-containing functional groups. ZnO crystalline peaks appeared at 31.7°, 34.4°, 36.2°, 47.5°, 56.5°, 64.1° and 67.8° corresponding to the lattice planes (100), (002), (101), (102), (110), (103), (112), and (201) (JCPDS No. 36–1451). High purity is confirmed by diffraction peaks associated with impurities that are not apparent in the XRD graph. The ZnO NPs integrated over GO sheets were investigated for their typical crystal size and composition using XRD analysis, which also provides an outline of the stated crystals’ states. The crystalline composition of the loaded ZnO NPs, which were free of flaws and other interfering elements, was revealed by the lattice structure and diffraction. Furthermore, narrower and more intense diffraction peaks suggest that the ZnO is well-crystalline, and bottom-of-the-peaks widening indicates smaller crystalline sizes. The Debye–Scherrer equation (*D = kλ/βcosθ*) was used to determine the size of ZnO NPs which resulted in an average size of ≈ 38 ± 2 nm.

**Fig. 6:**
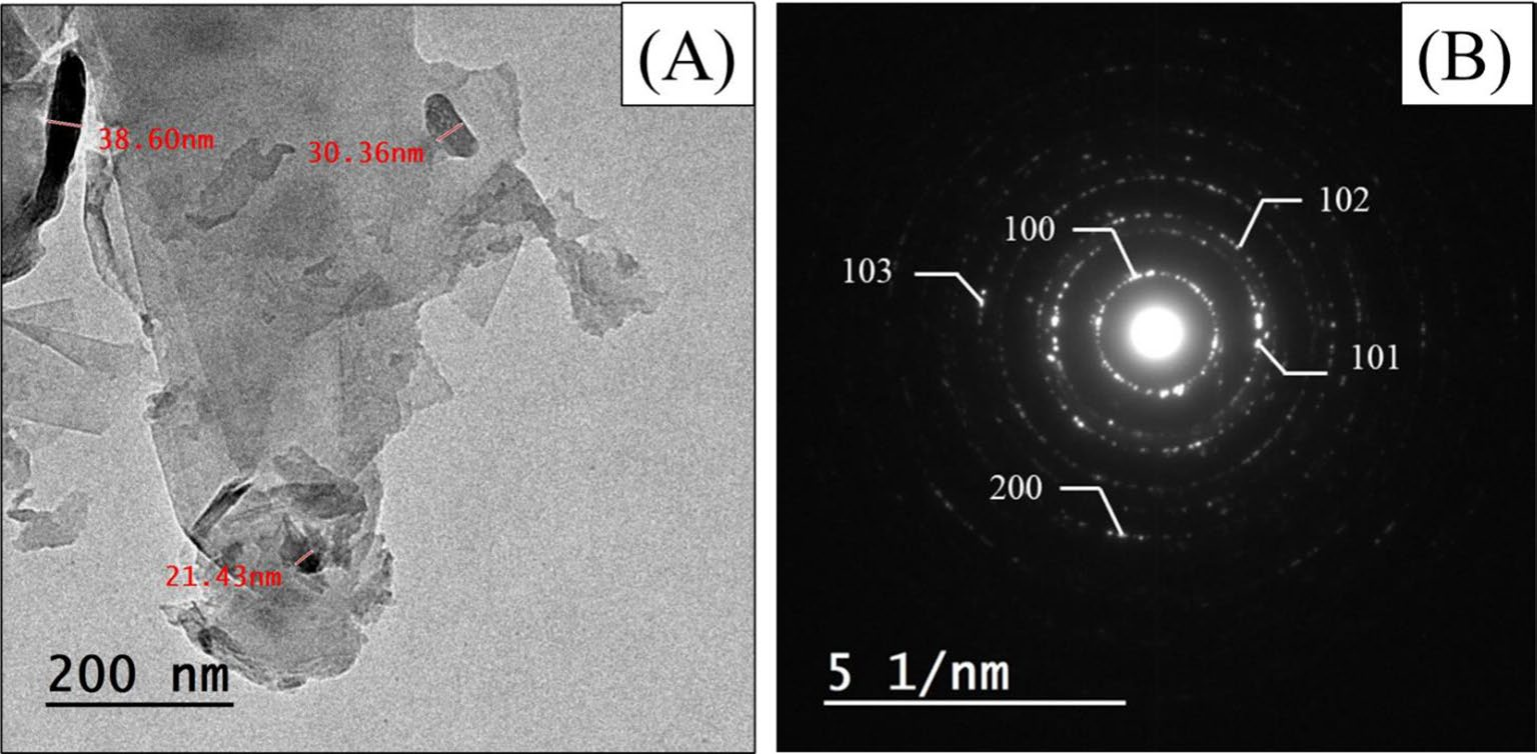
TEM micrograph; (**A**), and SADP pattern; (**B**), of GO/ZnO nanocomposite. Scale bar = 200 nm

TEM confirmed the successful synthesis of monolayered GO sheets as shown in Fig. 6A. Also, ZnO NPs appeared rod-shaped with an average size ranging from 30 to 40 nm, which is close to the XRD data. The SADP results confirmed the good crystallinity of the biosynthesized NPs as shown in Fig. 6B. The diffraction pattern of nano-colloidal particles appeared as lighted spots on the dark field.

**Fig. 7:**
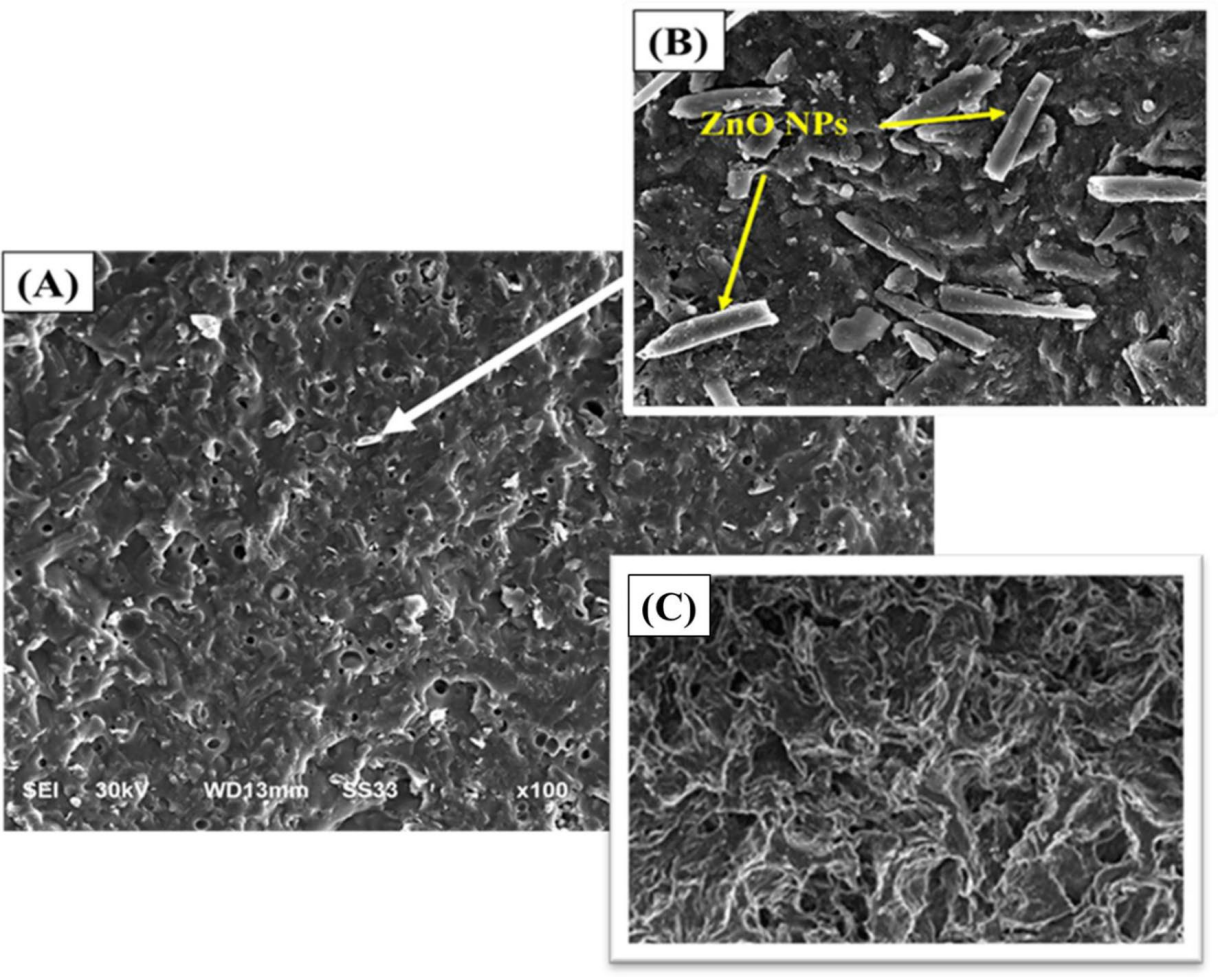
(**A**) SEM micrograph of CS/GO/ZnO cryogel. (**B**) A magnified part showing ZnO NPs rods decorated on the surface of the cryogel. (**C**) A magnified part showing the sponge-like structure with interconnected macropores of cryogel. Scale bar = 100 μm

SEM observations of CS/GO/ZnO cryogel were used to determine the dispersion, distribution, and particle size of GO/ZnO in the PVA matrix (Fig. 7A). According to mapping analysis, GO/ZnO showed good dispersion throughout the cryogel matrix. It also can be seen that particles are nano-sized. The average particle size of the ZnO NPs rods was found to be ≈ 37 ± 3.1 in length and 9 ± 2 nm in width (Fig. 7B). The CS/GO/ZnO cryogel appeared as a sponge-like structure (Fig. 7A) with interconnected macropores that resulted from the freezing of the reactants. The cryogel was porous with clear observable microchannels. The pores were heterogenous in their structure with diameters ranging between 10 and 60 μm or even greater.

### Antimicrobial activity studies

**Fig. 8:**
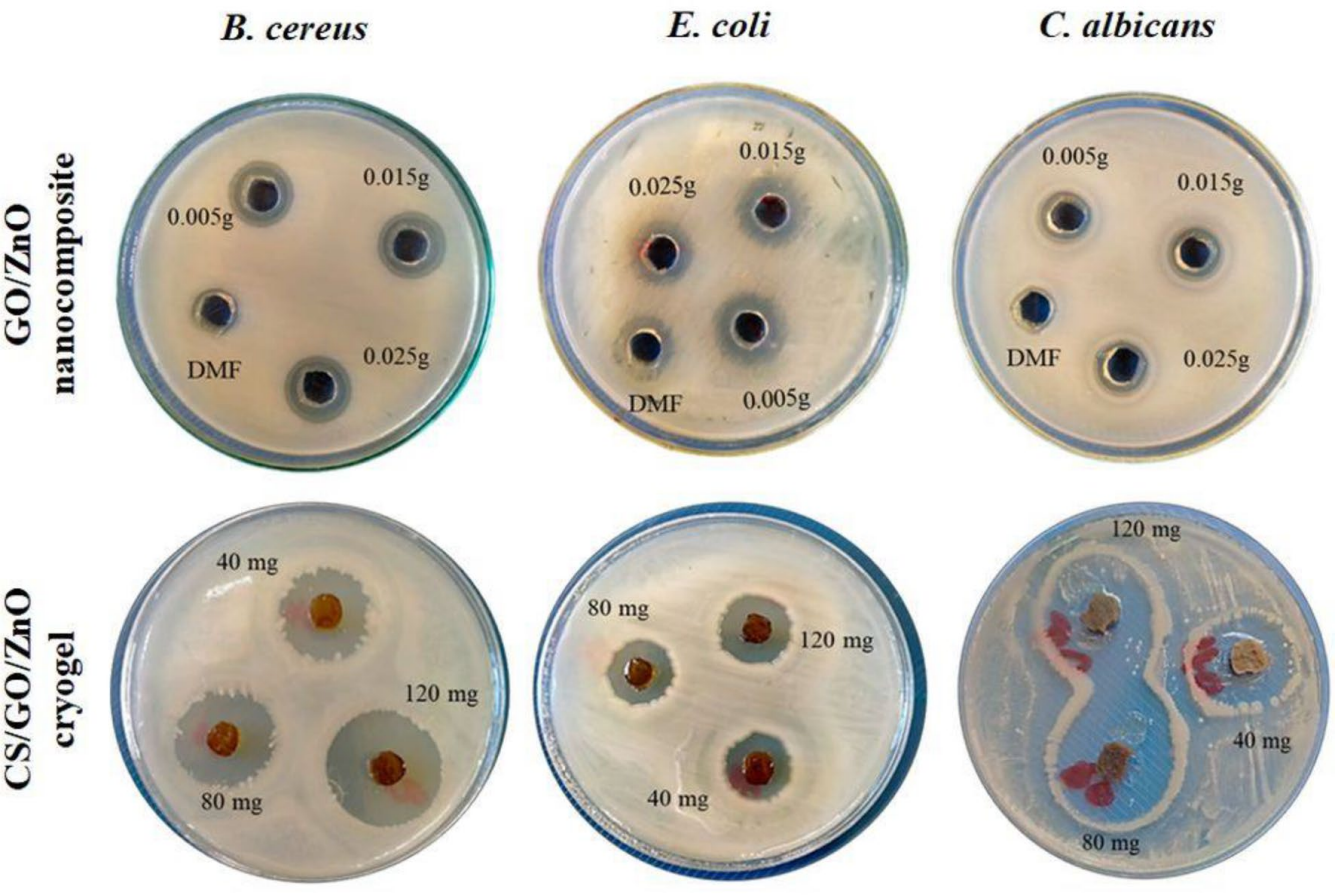
Antimicrobial activity of different concentrations of GO/ZnO nanocomposite and CS/GO/ZnO cryogel against the tested pathogenic microbial strains

GO/ZnO nanocomposite and CS/GO/ZnO cryogel showed a distinct antimicrobial activity against *B. cereus*,* E. coli*, and *C. albicans.* Significant differences were observed between the samples treated with different concentrations of GO/ZnO nanocomposite and standard antibiotics during the antimicrobial tests (Fig. 8; Table 1), and the diameter of the inhibition zone. Also, it was noted that CS/GO/ZnO cryogel discs (120 mg) revealed a superior antimicrobial action compared to the standard antimicrobial AMX. CS/GO/ZnO cryogel showed a stronger bactericidal effect against the Gram-positive bacterium *B. cereus* than the Gram-negative bacterium *E. coli*.

**Table 1:** Inhibition zone measurements of GO/ZnO nanocomposite and CS/GO/ZnO cryogel against the tested pathogenic microbial strains in comparison with AMX

Antimicrobial agents	Concentration, µg/ml	Zone of inhibition (mm, mean ± SD)*		
		B. cereus	E. coli	C. albicans
GO/ZnO nanocomposite	1%	15 ± 0^b^	20 ± 0.19^c^	12 ± 0.03^a^
	3%	15 ± 0^b^	21 ± 0.14^c^	14 ± 0^b^
	5%	15 ± 0^b^	19 ± 0.03^c^	13 ± 0^a^
CS/GO/ZnO cryogel	40 mg	21 ± 0.16^b^	17 ± 0.06^a^	24 ± 0.22^b^
	80 mg	23 ± 0.21^b^	18 ± 0.14^a^	32 ± 0.14^d^
	120 mg	28 ± 0.06^c^	19 ± 0.18^a^	26 ± 0.13^c^
AMX	120 mg	26.0 ± 1.00^c^	15.3 ± 0.46^a^	-
Miconazole	120 mg	-	-	11.3 ± 0.58^a^

*Means with common letters are not significantly different according to Duncan’s multiple range test (*P* < 0.05, *n* = 3)

**Fig. 9:**
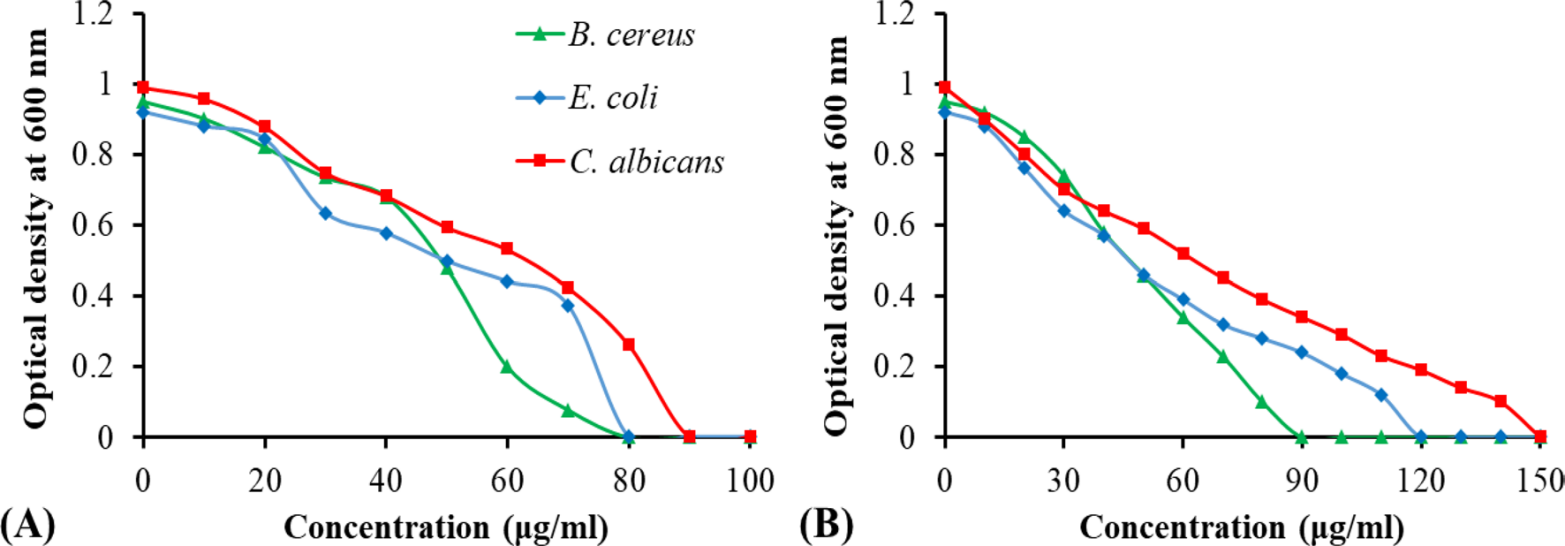
MIC of GO/ZnO nanocomposite; (**A**) against the tested pathogenic microbial strains compared to standard drugs; (**B**)

The MIC is the lowest antimicrobial agent concentration at which no detectable microbial growth is seen. Figure 9 displayed the MIC of GO/ZnO nanocomposite compared to AMX and miconazole. GO/ZnO nanocomposite had a similar ability to completely inhibit Gram-positive bacterium *B. cereus* and Gram-negative bacterium *E. coli* at 80 µg/ml compared to AMX (90 and 120 µg/ml, respectively). While it had MIC value activity against *C. albicans* reached 90 µg/ml compared to miconazole (150 µg/ml).

### Cytotoxicity assay

**Fig. 10:**
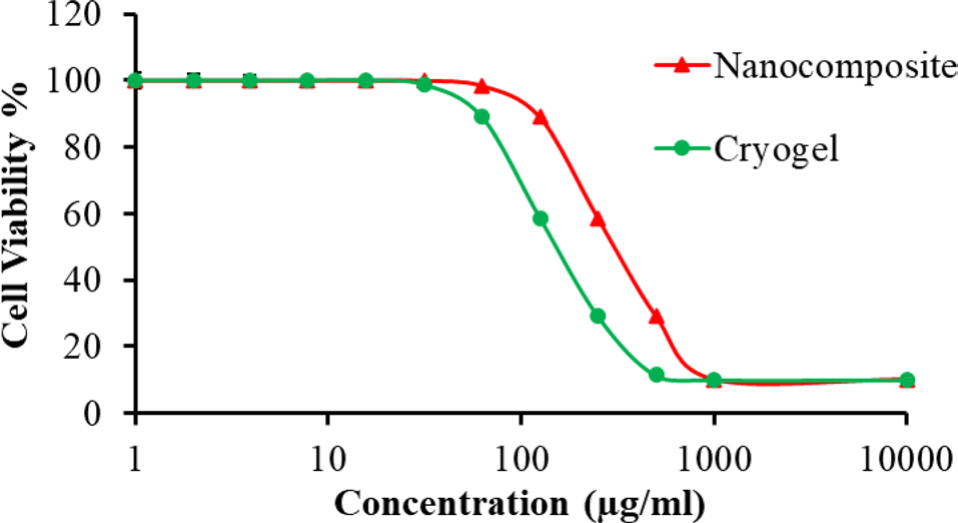
Cytotoxicity assay of CS/GO/ZnO alone or immobilized in the cryogel

CS/GO/ZnO composite or embedded in the cryogel did not show any cytotoxicity against WI-38 human fibroblast cell line up to the concentration of 31.25 µg/ml (Fig. 10). The concentration of the tested samples exhibited an inverse relationship with the percentage of cell viability. The estimated CC_50_ of the nanocomposite and cryogel was 209.90 ± 3.11 and 164.13 ± 2.32 µg/ml, respectively.

### Application of CS/GO/ZnO cryogel in water treatment

**Fig. 11:**
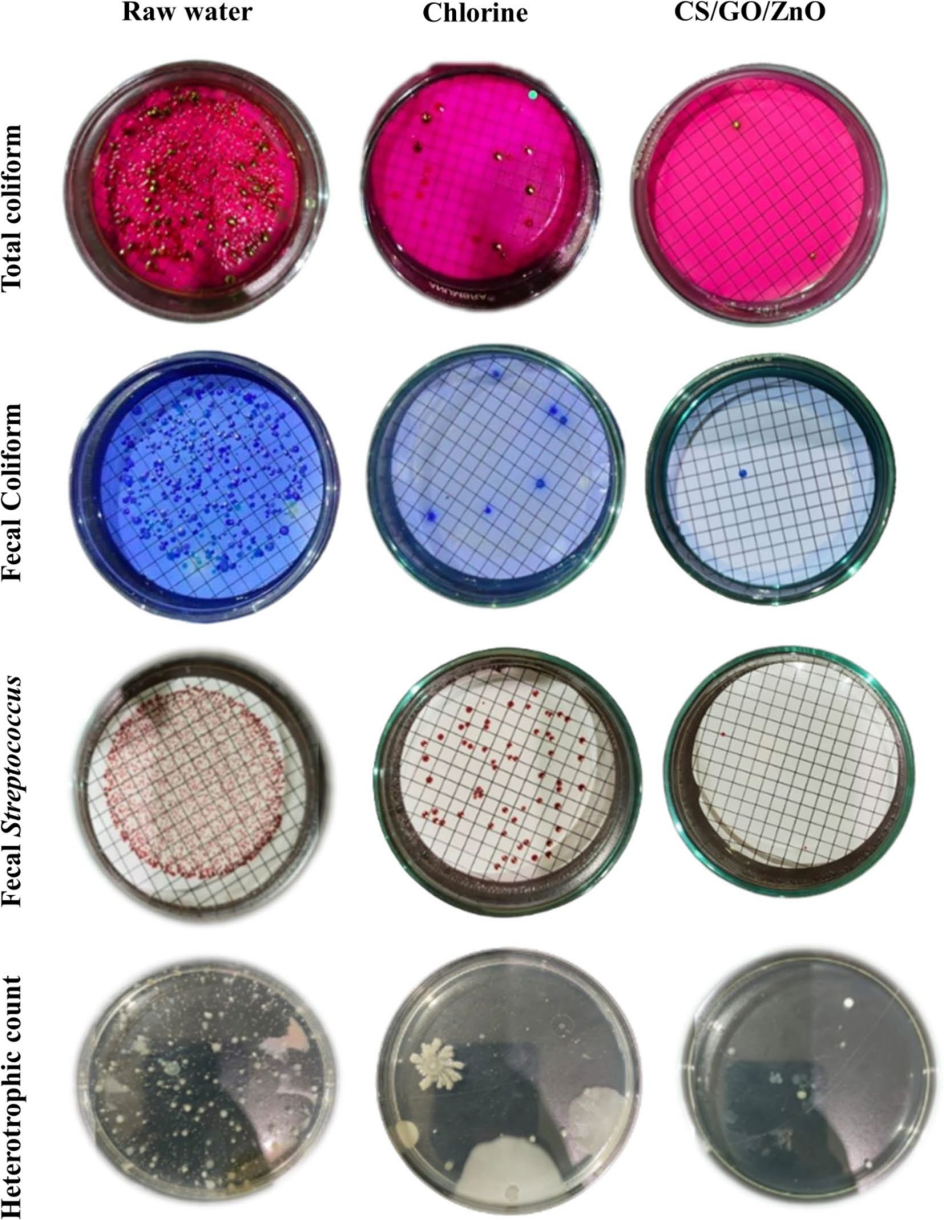
Total coliform, fecal coliform, fecal *Streptococcus*, and heterotrophic plate counts of CS/GO/ZnO cryogel-treated water samples compared to chlorine-treated samples

Different metals concentrations including Cu, Mn, Zn, Co, and Fe were estimated in the water samples before and after the treatment using CS/GO/ZnO cryogel (Table 2). The results revealed a decreasing metal concentration in the CS/GO/ZnO-treated samples. Cobalt was significantly decreased in this group compared to the chlorine group. Treatment with this nano-gel significantly decreased turbidity, total coliform, fecal coliform, fecal *Streptococcus*, and heterotrophic plate counts not only in comparison with the chlorine-treated samples but also with the raw water samples (Table 2; Fig. 11).

**Table 2:** Physicochemical and biological characteristics of untreated and treated water samples

Test	Standard limits		Current measurements*		
	Raw water	Drinking water	Raw water	Chlorine	CS/GO/ZnO cryogel
Turbidity (NTU)	-	1	6.9^c^	1.69^b^	0.58^a^
pH	7-8.5	6.5–8.5	8^c^	7.67^b^	6.5^a^
Conductivity	-	-	369^a^	378^b^	418^c^
TDS (ppm, 120⁰C)	500	1000	221^a^	232^b^	271^c^
Residual Al (ppm)	-	0.2	0	0.098^b^	0.015^a^
Cu (ppm)	1	2	UDL	UDL	UDL
Mn (ppm)	0.5	0.4	0.003	UDL	UDL
Zn (ppm)	1	3	0.022^c^	0.0064^a^	0.0172^b^
Co (ppm)	-	-	0.004^c^	0.002^b^	0.001^a^
Fe (ppm)	1	0.3	0.059^c^	0.049^a^	0.055^b^
Total coliform (CFU/100 ml)	1000	≤ 2	10,800^c^	4^a^	2^a^
Fecal coliform (CFU/100 ml)	-	< 1	660^c^	6^a^	1^a^
Fecal *Streptococcus* (CFU/100 ml)	-	< 1	800^c^	57^a^	2^a^
Heterotrophic plate count (CFU/1 ml)	-	≤ 50	4400^c^	140^a^	53^a^

*Means with common letters are not significantly different according to Duncan’s multiple range test (*P* < 0.05, *n* = 5), NTU; Nephelometric turbidity unit, UDL; Undetectable level

## Discussion

Microbial resistance to the microbicidal action of antibiotics and other antimicrobial agents had been recorded [49]. The production of extracellular matrix [62], biofilm adaptability [63], ion efflux pumps [64], electrostatic repulsion, and mutations are a few of the resistance mechanisms that have been recently discovered. The objective of this study is to find a new effective antimicrobial agent using a green, simple, and cost-effective method which can be used in drinking water treatment applications. Nanomaterials have recently emerged as a weapon against multi-drug resistant microbes. These nanomaterials have the potential to be employed as nano-drugs that combat resistant microbial strains by acting either independently or in concert with antimicrobial substances [65, 66]. Additionally, nanomaterials are employed as drug delivery systems that offer improved physicochemical properties and increased therapeutic efficacy [67]. Silver oxide, copper oxide, magnesium oxide, calcium oxide, zinc oxide, gold, silver, titanium, copper, zinc, and aluminum are among the most researched metal and metal oxide nanoparticles (NPs) against multidrug-resistant microbes [28]. Previously, several studies have reported that *B. subtilis*, *B. licheniformis*, and *B. cereus*, as bio-agents for the synthesis of ZnO NPs [68–70]. As an aerobic bacterium that is extensively found in soil and decomposing organic waste, *B. subtilis* is a harmless, safe, non-pathogenic microorganism and considered as one of the unique probiotic bacteria with a quick growth rate, few nutritional needs, effective secretion of several proteins and metabolites, and no ability to produce toxins [21, 50, 68]. *B. subtilis* is well-known in the industry for generating a wide variety of metabolites that act as bio-reducing agents in the biosynthesis of NPs [68]. The current study reported the ability of *B. subtilis* ATCC 6633 to extracellular biosynthesize ZnO NPs within 24 h. The biosynthesis of ZnO NPs was confirmed using UV-Vis spectroscopy and displayed an absorption peak at 358 nm due to the photoexcitation of electrons from the valance band to conduction band. Also, ZnO NPs have an absorption peak at 363 nm as reported by Hameed et al. [71] which was consistent with the current results. UV-Vis spectrum of GO shows an absorption peak at ≈ 235 nm. This peak can be attributed to the to π–π* transitions of the aromatic C = C bond of GO and n–π* transitions of C = O [72]. The biosynthesized GO /ZnO composite has an absorption peak around 358 nm almost as same as ZnO NPs indicating the existence of crystalline ZnO in the composite, which was similar to the previously published data [50].

The usage of NPs in various industrial and medical applications is frequently restricted by aggregation and agglomeration. To ascertain the NPs stability, FTIR and Zeta analyses were conducted. FTIR spectrum of ZnO, the peaks at 428 cm^− 1^ correspond to O-Zn-O stretching as documented by Zhang et al. [73]. FTIR spectrum of GO and GO/ZnO nanocomposite showed different characteristic bands of oxygen species such as carbonyl and carboxyl. Also, the alignment of ZnO on GO matrix was observed and the obtained results may imply that the ZnO NPs were successfully anchored on the surface of GO sheets [74]. Also, the presence of proteins in the production of NPs that could serve as stabilizing agents was established by measuring the FTIR spectra of ZnO NPs and GO/ZnO nanocomposite [52]. Based on the surface charge of the NPs, the zeta potential measurement indicates the colloidal stability of the synthesized NPs in solution. Zeta potential values that are either positive or negative show that the NPs are repulsed by one another, which could prevent their aggregation [75]. According to the zeta potential values, the stability of NPs was classified into four categories: very unstable (values within the range of ± 0–10 mV), somewhat stable (values within the range of ± 10–20 mV), stable (values within the range of ± 20–30 mV), and high stable (values beyond ± 30 mV) [76]. Thus, based on the obtained zeta potential value (-32.8 ± 5.7 mV), the synthesized GO/ZnO nanocomposite has a high stability. On the other hand, Rosnan et al. [52] prepared the ZnO-decorated GO nanocomposite which had a Zeta potential value of -28.23 mV. While Kim et al. [77] reported the absolute values for the zeta potential analysis were larger than 25, indicating the high colloidal stability of the fabricated NPs. The zeta potential value of biosynthesized ZnO NPs obtained by *Serratia nematodiphila* was measured as -33.4 mV, indicating that the NPs are highly stable in water [73]. Good stability and crystallinity of NPs were also confirmed by the XRD results. The XRD patterns displayed the successful biosynthesis of ZnO NPs and their anchor in GO sheets, which were matched with El-Nour et al. [78] and Mahdi et al. [38] results that used *B. subtilis* ATCC 6633 and *Bacillus* sp. PTCC1538, respectively. The absence of any additional peaks related to secondary, or impurity phases demonstrated the purity of the prepared NPs. After the oxidation of Gt to GO, the XRD pattern of GO showed the disappearance of Gt diffraction peak at 2*θ* = 26.35º which confirmed the fabrication of GO [79]. The successful exfoliation of GO sheets as a result of ZnO NPs being anchored over their surface may account for the reduced intensity of the GO peak in the nanocomposite [80].

Based on the TEM micrographs, a very thin sheet of 2D layered GO was fabricated and embellished with ZnO NPs. Additionally, ZnO NPs were shown to be significantly intercalated between GO sheets, supporting the synthesis of GO/ZnO. Diffraction rings in the SADP pattern clearly show that the samples are polycrystalline, and all of them are allocated to an orthorhombic structure, which is consistent with the findings of the XRD. This suggests that the particles are GO/ZnO nanocrystals in a single phase which matched with the Jayachandiran et al. [81] data. ZnO NPs of this study were significantly smaller than ZnO NPs prepared by Mahdi et al. [38] using *Bacillus* sp. (99 nm). Also, the biosynthesized ZnO nano-rods using *Xanthomonas campestris* had a diameter of 300 nm as reported by Mahdi et al. [82]. While Saleh et al. [83] reported the diameter of ZnO NPs ranged from 22 to 59 nm that biosynthesized using *B. subtilis*. The surface morphology of the GO/ZnO cryogel was studied using SEM investigation, demonstrating pore development throughout the cryogel processing. The separation of the liquid and solid phases within the cryogel was what caused the pores formation. The liquid phase first changed into ice crystals, which progressively enlarged until they come into contact with one another [84]. The ice crystals in the hydrogel evaporated during the freeze-drying process, leaving pores in the cryogel structure. Furthermore, the liquid phase prevented the cryogel from collapsing and facilitates the formation of its pore network. It is observed that the hydrogel structure changed and become more porous when CS is present in the PVA cryogel. The hydrogel as a catalyst support provides ideal conditions for the uniform dispersion of ZnO NPs and GO while preserving the structure of the PVA matrix. A previous study has shown that the addition of CS increased the pores formation in the hydrogel and helped in their distribution more uniformly [85]. ZnO/PVA/CS and plasticizers with pore size approximately 8 μm was conducted by Vicentini et al. [86]. Another study prepared PVA/TiO_2_ gel resulted in an average pore size 20 μm and 35 μm, PVA/Fe_2_O_3_ with 25 to 32 μm and pure PVA gel with a wide range of pore sizes ranging from 10 to 75 μm [87]. Khodaee et al. [88] synthesized PVA/reduced GO (PVA/rGO) hydrogel with pore sizes of range between 7 and 45 μm. The hydrogel as a catalyst support provides ideal conditions for the uniform dispersion of ZnO NPs and GO while preserving the structure of the PVA matrix.

It is well known that metals and metals oxides are recognizable strong antimicrobial agents against several pathogenic microbial cells [30, 35, 89]. Recently variety of nano-structural materials especially biologically synthesized have been proven promising antimicrobial properties, this suffices for a larger area of interactions with biological systems [36]. In this study, green synthesized ZnO NPs, GO/ZnO and CS/GO/ZnO cryogel were tested against different bacterial strains such as *B. cereus* and *E. coli* in addition to *C. albicans* as a pathogenic yeast. All concentrations of GO/ZnO exhibited promising antimicrobial activity against *E. coli* and *C. albicans* compared to conventional antimicrobial agents, AMX and miconazole. GO/ZnO containing 3% ZnO NPs had the highest antibacterial impact on *E. coli* with inhibition zone of 21 ± 0.14 mm among all the tested materials. On the other hand, GO/ZnO containing 1% ZnO NPs showed the lowest influence towards *C. albicans* with inhibition zone measuring 12 ± 0.03 mm. This corresponds with Bhaisare [90] study which confirmed the higher activity of GO/ZnO against Gram-negative (*E. coli*) than Gram-positive (*S. aureus*). Elbasuney et al. [91] confirmed the strong antimicrobial activity of reduced GO/ZnO against *E. coli* (21.7 ± 0.58 mm), *B. subtilis* (38.5 ± 0.50 mm), and *C. albicans* (19.3 ± 0.58 mm). It was also noted that the concentration of ZnO NPs in the composite played a significant role in the activity of the prepared nanocomposite [92, 93]. The adsorption capability of the GO/ZnO decreases with ZnO NPs additions more than 3%. The decrease in GO surface area as ZnO NPs percentage increased can be used to explain the decline in adsorption effectiveness at higher ZnO NPs percentages. Furthermore, by creating a new functional group between the GO layers, an excess of ZnO NPs may also cause ZnO NPs and GO sheets to aggregate, which may make the active sites less accessible for adsorption [94]. Gram-negative bacteria were more susceptible to the antibacterial action of GO/ZnO than Gram-positive bacteria, which may have resulted from the differences in the bacterial cell wall structures. When compared to Gram-negative bacteria, the detrimental effects of GO/ZnO were lessened by the thick walls and high levels of peptidoglycan present in the cell walls of Gram-positive bacteria [95].

The MIC values of the prepared material were measured and determined as 80 µg/ml against *E. coli* and *B. cereus* and 90 µg/ml against *C. albicans*. Other studies had reported an efficient antibacterial action of the GO/ZnO nanocomposite with MIC values ranged between 25 and 100 µg/ml [96–98]. Results showed also that the antimicrobial action increased by increasing the concentration of ZnO NPs in GO/ZnO. A previous study by Brandão et al. [99] showed that the incorporation of 2–5 wt% of ZnONP in composites could endow antimicrobial activity to the composite without jeopardizing their physicochemical properties. Nevertheless, an overabundance of ZnO NPs caused them to aggregate and wrap around the GO sheet, perhaps reducing the GO’s active sites and exhibiting an inhibition rate that is lower or comparable to a lower concentration [98].

The prevailing consensus is that the antibacterial activity of NPs and their size are inversely correlated. That smaller NPs have a higher surface area to volume ratio and, therefore, more bioactivities could be one explanation for this observation [100]. According to the current study TEM and SEM results, the prepared NPs had a small size range which may illustrate their strong antimicrobial behavior. Stanković et al. [101] reported that the antibacterial activity of ZnO NPs with an average diameter around 30 nm against *S. aureus* and *E. coli* had the greatest antibacterial activity compared with other larger sizes.

Both the biological properties and stiffness of the composite can be enhanced at the same time by the biopolymer that contains the NPs, and vice versa. As a result, the composite’s options and usage frequency are increased. Due to their biodegradability, bio-nanocomposites and NPs provide ecological sustainability and allow for the medication’s eco-compatibility. The improved qualities of medicine and the resulting favorable effects on the environment are the result [102]. PVA is becoming more and more well-known due to its remarkable ability to produce films, environmental friendliness, and biocompatibility. PVA is an atactic semi-crystalline polymer with emulsifying and adhesive properties. It is resistant to solvents, oil, and grease because of its chemical stability. PVA is hydrophilic and has a high density of functional groups that are reactive. In addition, it has a high tensile strength, flexibility, and great oxygen and smell barrier properties [103]. GO/ZnO hydrogel delivered antibacterial agents, such as GO/ZnO nanosheets, ZnO NPs and Zn^2+^ ions, to the surrounding agar, resulting in the inhibition zones against the tested microbial strains which matched with Le et al. [104] reports. Abd El-Mohdy et al. [105] recorded the antimicrobial activity of pure PVA hydrogel against *B. subtilis* and *C. albicans*. Also, Chowdhuri et al. [93] synthesized GO/CS/ZnO which showed an antibacterial potency towards both Gram-negative bacteria and Gram-positive. The PVA/ZnO nanofibers were synthesized by Khan et al. [106], who also found dose-dependent ZnO NP content and effective antibacterial action against *S. aureus* and *E. coli*. Gutha et al. [107] and Abdeen et al. [108] prove the uniqueness of a capping agent towards surface area and antibacterial activity by synthesis of a CS/PVA/ZnO nanocomposite with a much higher antimicrobial activity against Gram-positive and Gram-negative bacteria due to the presence of ZnO NPs. There are different illustrations for the antimicrobial action of GO composites. According to the most widely accepted theory, when microbial cells were deposited on graphene surfaces, membrane tension developed and may have harmed the cell membrane structure. Notwithstanding the fact that no superoxide anions were found, oxidative stress that is not dependent on reactive oxygen species (ROS) may also aid in the inactivation of bacteria in addition to membrane stress, as demonstrated by glutathione oxidation [109]. Similar to this, studies by Krishnamoorthy et al. [92] showed that GO produced ROS, and that these free radicals in turn led to bacterial cell lipid peroxidation in strains of *E. coli* and *B. subtilis*. According to Tu et al. [110], the strong dispersion interactions between graphene and lipid molecules allow graphene nanosheets to penetrate cell membranes and extract substantial amounts of phospholipids. Though superoxide anions produced by GO and rGO were reported to be capable of inactivating *P. aeruginosa* by Gurunathan et al. [111].

CS/GO/ZnO nanocomposite and cryogel were tested for their cytotoxic activity against WI-38 human fibroblast cell line using MTT assay. When the concentrations of CS/GO/ZnO nanocomposite and cryogel decrease, the cell viability increases until they reach the CC_50_. The CC_50_ values were found to be 209.90 ± 3.11 and 164.13 ± 2.32 µg/ml for CS/GO/ZnO nanocomposite and cryogel, respectively. High CC_50_ confirmed their safety. It was obvious that the CC_50_ of CS/GO/ZnO nanocomposite was significantly higher than that of cryogel. These results indicated that the nanocomposite had the lower significant cytotoxicity against WI-38 cell line. This difference might be due to the presence of glutaraldehyde in the synthesis of cryogel [112]. Cytotoxic activity of nanocomposites and cryogels on different human cells is essential for their industrial, agricultural, and biomedical applications. Higher lethal doses and lower CC_50_ values were observed in several studies against human cells and other animal cells. Hemati et al. [113] prepared liposomal sulforaphane-loaded PVA/polyethylene glycol hydrogels and reported its CC_50_ of 107.2 µg/ml against human skin fibroblasts. Also, PVA-Ag and CS-Ag nanocomposites films showed low cytotoxic activities against Huh-7 liver cells and CC_50_ values of 185.07 µg/ml and 119.85 µg/ml, respectively as reported by Abdallah et al. [114]. While Abed Shlaka et al. [115] studied the activity of the modified chitosan/PVA/Au against rat embryonic fibroblasts (REF) and recorded its CC_50_ as 83.92 µg/ml.

CS/GO/ZnO cryogel was also applied for water treatment as disinfectant and anti-biofouling agents. Different bacterial inactivation occurred after the treatment of raw water samples with the prepared cryogel including total coliform, fecal coliform, fecal *Streptococcus*, and heterotrophic plate counts. Our results showed 99.98%, 99.8%, and 99.75% efficiency for eliminating total coliform, fecal coliform, and fecal *Streptococcus*, respectively, within 15 min at room temperature (25 °C) and 90 rpm, indicating that CS/GO/ZnO cryogel can be applied to inactivate a variety of bacteria. Additionally, the agar plates present the inactivation results of heterotrophic plate count with an efficiency reached to 98.79%, revealing the effectiveness of water disinfection of CS/GO/ZnO cryogel. CS/GO/ZnO cryogel could be applied as a promising and competitive antibacterial material compared to the current traditional materials based on its total disinfection efficacy at room temperature and without energy input. On the other hand, Guo et al. [116] studied the freeze-dried CS/3,4-dihydroxybenzaldehyde hydrogels capacity for rapid water disinfection and recorded its ability for a rapid inactivation of > 99% bacteria in 60 min at ambient conditions. Chen et al. [117] studied the antibacterial action of PVA hydrogels reinforced with graphene-doped ZnO nanoplates and reported that graphene and ZnO improved the swelling degree, tensile property, and dye adsorption capacity of PVA hydrogel moreover it endowed the hydrogel antibacterial characteristics. While Zeng et al. [118] documented that the Ag/rGO hydrogel showed inactivation rates against *E. coli* and total coliform reached to > 94.51% and 99.71%, respectively during the disinfection of natural water samples. Zhang et al. [119] reported the excellent antibiofouling activity of the rGO/ZnO/polyethersulfone membrane against Gram-negative *P. aeruginosa*. The crosslinking between three antibacterial motifs (ZnO NPs, GO, and CS) and PVA cryogel increased their chemical stability. Furthermore, there are minimal oxidation effects and both motifs are comparatively inert in water. As a result, they are not expected to leave behind residues or produce hazardous byproducts or have leftover residues. After removing the CS/GO/ZnO cryogel discs with a colander spoon, we examined the presence of chemical residues and NPs leaching directly. The results of CS/GO/ZnO cryogel-treated water had a very small rise in TDS. While results for residual Al, Cu, Mn, Zn, Co, and Fe measurements confirmed there are no chemical impurities remaining after the disinfection test which agreed with Guo et al. [116] records. Overall, the results showed that all contaminants and impurities (turbidity, alkalinity, residual Al, Cu, Mn, Zn, Co and total coliform) in raw water samples were reduced to a level matched with the drinking water standards of the WHO and the EWQS [59, 120]. Therefore, the CS/GO/ZnO cryogel has the potential to enhance or replace the current traditional disinfection methods.

The nature of the materials determines the adsorption of heavy metals onto macroporous polymers, which usually includes several pathways. The chemistry of the aqueous phase plays a major role in the effective removal of hazardous metal ions. Heavy metals have been removed from water using a variety of techniques, including membrane separations, ion exchange and adsorption, chemical precipitation, coagulation, and bioremediation [121]. Adsorbents for the removal of heavy metals have included synthetic polymers (hydrogels, cryogels), as well as biopolymers [122]. Cryogels are polymeric materials that use electrostatic forces to remove ionic pollutants from water. Also, they are more elastic and have a far higher water retention capacity, whose structures are mostly stiff. Certain functional groups in the structure, such -OH, -NH_2_, -COOH, and -CONH_2_, are improving the removal of chemicals from water due to their ability to be absorbed both into the swollen 3D cryogel system and onto the outside surface [123]. Additionally, the open-ended holes in cryogels, as opposed to the closed-end pores in traditional porous materials, help to promote rapid diffusion [124].

The current study highlighted a low-cost method for environmental applications that can rival the existing ones that use expensive materials. Cryogel-based techniques can be worth the extra cost if they improve water treatment by being able to handle particularly persistent contaminants and dangerous substances that cannot be removed with current traditional methods. A tap or small-scale treatment plant application is possible for small volume cryogels, according to recent research and evaluation on the materials [125]. The unique properties of polymers (PVA and CS), such as their affordable price, chemical versatility, compatibility, degradability, non-toxicity, and manufacturing facility, make them ideal for the fabrication of cryogel [126]. In addition, the cost-effective microbial synthesis of ZnO NPs and cryogelation processes is more efficient, consumes no energy, and beneficial than other processes [127]. Some good things about macroporous polymeric structures include their high-water content, non-toxicity, efficiency, ease of storage, and reusability [84]. The inexpensive cost of raw materials combined with the significant advantages of the CS/GO/ZnO suggests it as feasible choice for water treatment and purification.

## Conclusions

An extracellular biosynthesis of ZnO NPs was prepared using *B. subtilis* ATCC 6633, decorated on GO sheets then fabricated a CS/GO/ZnO PVA cryogel as a final product.

Different physicochemical investigations for the prepared materials were studied such as UV-Vis spectroscopy, FTIR, Zeta potential, XRD, SADP, and SEM. The synthesized GO/ZnO and CS/GO/ZnO PVA cryogel exhibit potent antimicrobial activities in a dose-dependent manner. Results revealed that the Gram-positive, Gram-negative bacteria, and unicellular fungus were sensitive toward GO/ZnO and CS/GO/ZnO cryogel at low concentrations, with MIC values of 80, 80, 90 µg/ml, respectively. The current study provided also a simple route to endow a cryogel with antibacterial and antibiofouling capacities for water. By embedding ZnO NPs and CS onto the GO backbone, enabling inactivation of bacteria in raw water samples. When CS/GO/ZnO cryogel discs applied directly in raw water treatment, they showed a rapid inactivation of ≈ 99% bacteria within 15 min at room temperature without any energy consumption. Real river, Nile River, Egypt, water samples were used to investigate the efficiency of CS/GO/ZnO cryogel on water purification and bacterial inactivation. CS/GO/ZnO cryogel revealed interesting results in decreasing contaminants and impurities (turbidity, residual Al, Cu, Mn, Zn, Co, bacteria) in water samples suggesting it as a significant environmentally friendly, nontoxic potential agent for practical water treatment at the household or community scale. Furthermore, the toxicity, antimicrobial activity and nanoremediation action of CS/GO/ZnO cryogel needs to be studied in vivo with an animal model. In addition, limitations of fabricated cryogel when it comes to a large-scale application, stability, reusability, and recycling tests should be also investigated in future studies.

## Data Availability

No datasets were generated or analysed during the current study.

## References

[1] Szálkai K. Water-borne diseases. In: Romaniuk S, Thapa M, Marton P, editors. The Palgrave encyclopedia of global security studies. Cham, Switzerland: Springer International Publishing; 2019. pp. 1540–6. 10.1007/978-3-319-74319-6.

[2] Edokpayi JN, Odiyo JO, Durowoju OS. Impact of wastewater on surface water quality in developing countries: A case study of South Africa. In: Water quality. Vienna, Austria: IntechOpen; 2017. pp. 401–416. 10.5772/66561

[3] WHO (World Health Organization). Drinking-water. https://www.who.int/news-room/fact-sheets/detail/drinking-water. Accessed 15 Nov 2021.

[4] Prüss-Üstün A, Bos R, Gore F, editors. Safer water, better health: costs, benefits and sustainability of interventions to protect and promote health. Geneva, Switzerland: WHO; 2008. http://whqlibdoc.who.int/publications/2008/9789241596435_eng.pdf

[5] Huertas E, Salgot M, Hollender J, Weber S, Dott W, Khan S, Schaefer A, Messalem R, Bis B, Aharoni A, Chikurel H. Key objectives for water reuse concepts. Desalination. 2008;218:120–31. 10.1016/j.desal.2006.09.032.

[6] Richardson SD, Postigo C. Drinking water disinfection by-products. Emerging organic contaminants and human health. In: Barceló D, editor. Emerging organic contaminants and human health. The handbook of environmental chemistry. Berlin, Heidelberg: Springer; 2011. pp. 93–137. 10.1007/698_2011_125.

[7] Sun X, Chen M, Wei D, Du Y. Research progress of disinfection and disinfection by-products in China. J Environ Sci. 2019;81:52–67. 10.1016/j.jes.2019.02.003. 10.1016/j.jes.2019.02.003 30975330

[8] Gelete G, Gokcekus H, Ozsahin DU, Uzun B, Gichamo T. Evaluating disinfection techniques of water treatment. Desalin Water Treat. 2020;177:408–15. 10.5004/dwt.2020.25070.

[9] He K, Chen G, Zeng G, Chen A, Huang Z, Shi J, Huang T, Peng M, Hu L. Three-dimensional graphene supported catalysts for organic dyes degradation. Appl Catal B: Environ. 2018;228:19–28. 10.1016/j.apcatb.2018.01.061.

[10] Kang J, Zhang H, Duan X, Sun H, Tan X, Liu S, Wang S. Magnetic Ni-Co alloy encapsulated N-doped carbon nanotubes for catalytic membrane degradation of emerging contaminants. J Chem Eng. 2019;362:251–61. 10.1016/j.cej.2019.01.035.

[11] Yang Q, Peng J, Xiao H, Xu X, Qian Z. Polysaccharide hydrogels: functionalization, construction and served as Scaffold for tissue engineering. Carbohydr Polym. 2022;278:118952. 10.1016/j.carbpol.2021.118952. 34973769 10.1016/j.carbpol.2021.118952

[12] Bashir S, Hina M, Iqbal J, Rajpar AH, Mujtaba MA, Alghamdi NA, Wageh S, Ramesh K, Ramesh S. Fundamental concepts of hydrogels: synthesis, properties, and their applications. Polymers. 2020;12:2702. 10.3390/polym12112702. 33207715 10.3390/polym12112702 PMC7697203

[13] Sahiner N, Demirci S. PEI-based hydrogels with different morphology and sizes: Bulkgel, microgel, and cryogel for catalytic energy and environmental catalytic applications. Eur Polym J. 2016;76:156–69. 10.1016/j.eurpolymj.2016.01.046.

[14] Thakur S, Sharma B, Verma A, Chaudhary J, Tamulevicius S, Thakur VK. Recent approaches in guar gum hydrogel synthesis for water purification. Int J Polym Anal Ch. 2018;23(7):621–32. 10.1080/1023666X.2018.1488661.

[15] Lupu A, Gradinaru LM, Gradinaru VR, Bercea M. Diversity of bioinspired hydrogels: from structure to applications. Gels. 2023;9(5):376. 10.3390/gels9050376. 37232968 10.3390/gels9050376 PMC10217308

[16] Salomé Veiga A, Schneider JP. Antimicrobial hydrogels for the treatment of infection. Pep Sci. 2013;100:637–44. 10.1002/bip.22412. 10.1002/bip.22412 PMC3929057 24122459

[17] Joshi Navare K, Eggermont LJ, Rogers ZJ, Mohammed HS, Colombani T, Bencherif SA. Antimicrobial hydrogels: key considerations and engineering strategies for biomedical applications. In: Li B, Moriarty T, Webster T, Xing M, editors. Racing for the surface: pathogenesis of implant infection and advanced antimicrobial strategies. Cham: Springer; 2020. pp. 511–42. 10.1007/978-3-030-34475-7_22.

[18] Abou-Dobara MI, Kamel MA, El-Sayed AKA, El-Zahed MM. Antibacterial activity of extracellular biosynthesized iron oxide nanoparticles against locally isolated β-lactamase-producing *Escherichia coli* from Egypt. Discov Appl Sci. 2024;6:113. 10.1007/s42452-024-05770-z.

[19] El–Zahed MM, Eissa MS, Moawed EA, El Sadda RR. Application of thiourea polyurethane foam/zinc oxide nanocomposite for anticancer effects and antimicrobial potential. Discov Appl Sci. 2024;6:112. 10.1007/s42452-024-05750-3.

[20] Simeonidis K, Mourdikoudis S, Kaprara E, Mitrakas M, Polavarapu L. Inorganic engineered nanoparticles in drinking water treatment: a critical review. Environ Sci: Water Res Technol. 2016;2:43–70. 10.1039/C5EW00152H.

[21] El-Zahed AA, Khalifa ME, El-Zahed MM, Baka ZA. Biological synthesis and characterization of antibacterial manganese oxide nanoparticles using *Bacillus subtilis* ATCC6633. SJDFS. 2023;13:79–87. 10.21608/sjdfs.2023.242279.1136.

[22] Saqib S, Faryad S, Afridi MI, Arshad B, Younas M, Naeem M, Zaman W, Ullah F, Nisar M, Elgorban AS, Syed AM, Elansary A, El-Abedin HO. Bimetallic assembled silver nanoparticles impregnated in *Aspergillus fumigatus* extract damage the bacterial membrane surface and release cellular contents. Coatings. 2022;12:1505. 10.3390/coatings12101505.

[23] Mendes CR, Dilarri G, Forsan CF, Sapata VD, Lopes PR, de Moraes PB, Montagnolli RN, Ferreira H, Bidoia ED. Antibacterial action and target mechanisms of zinc oxide nanoparticles against bacterial pathogens. Sci Rep. 2022;12:2658. 10.1038/s41598-022-06657-y. 35173244 10.1038/s41598-022-06657-y PMC8850488

[24] Mohamed AA, Abu-Elghait M, Ahmed NE, Salem SS. Eco-friendly mycogenic synthesis of ZnO and CuO nanoparticles for *in vitro* antibacterial, antibiofilm, and antifungal applications. Biol Trace Elem Res. 2021;199:2788–99. 10.1007/s12011-020-02369-4. 32895893 10.1007/s12011-020-02369-4

[25] Sirelkhatim A, Mahmud S, Seeni A, Kaus NH, Ann LC, Bakhori SK, Hasan H, Mohamad D. Review on zinc oxide nanoparticles: antibacterial activity and toxicity mechanism. Nanomicro Lett. 2015;7:219–42. 10.1007/s40820-015-0040-x. 30464967 10.1007/s40820-015-0040-x PMC6223899

[26] Anitha S, Brabu B, Thiruvadigal DJ, Gopalakrishnan C, Natarajan TS. Optical, bactericidal and water repellent properties of electrospun nano-composite membranes of cellulose acetate and ZnO. Carbohydr Polym. 2012;87:1065–72. 10.1016/j.carbpol.2011.08.030. 10.1016/j.carbpol.2013.05.003 24066357

[27] Saqib S, Nazeer A, Ali M, Zaman W, Younas M, Shahzad A, Sunera, Nisar M. Catalytic potential of endophytes facilitates synthesis of biometallic zinc oxide nanoparticles for agricultural application. Biometals. 2022;35:967–85. 10.1007/s10534-022-00417-1. 35834149 10.1007/s10534-022-00417-1

[28] Wahid F, Zhong C, Wang HS, Hu XH, Chu LQ. Recent advances in antimicrobial hydrogels containing metal ions and metals/metal oxide nanoparticles. Polymers. 2017;9(12):636. 10.3390/polym9120636. 30965938 10.3390/polym9120636 PMC6418809

[29] Adams LK, Lyon DY, Alvarez PJ. Comparative eco-toxicity of nanoscale TiO_2_, SiO_2_, and ZnO water suspensions. Water Res. 2006;40:3527–32. 10.1016/j.watres.2006.08.004. 17011015 10.1016/j.watres.2006.08.004

[30] Li Q, Mahendra S, Lyon DY, Brunet L, Liga MV, Li D, Alvarez PJ. Antimicrobial nanomaterials for water disinfection and microbial control: potential applications and implications. Water Res. 2008;42:4591–602. 18804836 10.1016/j.watres.2008.08.015

[31] Pulit-Prociak J, Chwastowski J, Kucharski A, Banach M. Functionalization of textiles with silver and zinc oxide nanoparticles. Appl Surf Sci. 2016;385:543–53. 10.1016/j.apsusc.2016.05.167.

[32] Jones N, Ray B, Ranjit KT, Manna AC. Antibacterial activity of ZnO nanoparticle suspensions on a broad spectrum of microorganisms. FEMS Microbiol Lett. 2008;279(1):71–6. 10.1111/j.1574-6968.2007.01012.x. 18081843 10.1111/j.1574-6968.2007.01012.x

[33] Zhang LL, Chen B, Xie LL, Li ZF. Study on the antimicrobial properties of ZnO suspension against Gram-positive and Gram-negative bacteria strains. Adv Mater Res. 2012;393:1488–91. 10.4028/www.scientific.net/AMR.393-395.1488.

[34] Narayanan PM, Wilson WS, Abraham AT, Sevanan M. Synthesis, characterization, and antimicrobial activity of zinc oxide nanoparticles against human pathogens. BioNanoScience. 2012;2:329–35. 10.1007/s12668-012-0061-6.

[35] Espitia PJ, Soares ND, Coimbra JS, de Andrade NJ, Cruz RS, Medeiros EA. Zinc oxide nanoparticles: synthesis, antimicrobial activity and food packaging applications. Food Bioproc Tech. 2012;5:1447–64. 10.1007/s11947-012-0797-6.

[36] Agarwal H, Kumar SV, Rajeshkumar S. A review on green synthesis of zinc oxide nanoparticles–An eco-friendly approach. Resour-Effic Technol. 2017;3:406–13. 10.1016/j.reffit.2017.03.002.

[37] El-Nour AT, Abou-Dobara MI, El-Sayed AK, El-Zahed MM. Antibacterial activity of optimized extracellular biosynthesized zinc oxide nanoparticles using *Corynebacterium* Sp. ATCC 6931 SJDFS. 2023;13(3):63–70. 10.21608/sjdfs.2023.231788.1129.

[38] Mahdi ZS, Talebnia Roshan F, Nikzad M, Ezoji H. Biosynthesis of zinc oxide nanoparticles using bacteria: a study on the characterization and application for electrochemical determination of bisphenol A. Inorg Nano-Met Chem. 2021;51(9):1249–57. 10.1080/24701556.2020.1835962.

[39] Al-Kordy HM, Sabry SA, Mabrouk ME. Statistical optimization of experimental parameters for extracellular synthesis of zinc oxide nanoparticles by a novel haloalaliphilic *Alkalibacillus* sp. W7. Sci Rep. 2021;11:10924. 10.1038/s41598-021-90408-y. 34035407 10.1038/s41598-021-90408-y PMC8149680

[40] Wang Y, Ling C, Chen Y, Jiang X, Chen GQ. Microbial engineering for easy downstream processing. Biotechnol Adv. 2019;37:107365. 10.1016/j.biotechadv.2019.03.004. 30851362 10.1016/j.biotechadv.2019.03.004

[41] Saqib S, Zaman W, Ullah F, Majeed I, Ayaz A, Hussain-Munis MF. Organometallic assembling of chitosan-Iron oxide nanoparticles with their antifungal evaluation against Rhizopus oryzae. Appl Organomet Chem. 2019;33:e5190. 10.1002/aoc.5190.

[42] Saqib S, Zaman W, Ayaz A, Habib S, Bahadur S, Hussain S, Muhammad S, Ullah F. Postharvest disease inhibition in fruit by synthesis and characterization of Chitosan iron oxide nanoparticles. ISBAB. 2020;28:101729. 10.1016/j.bcab.2020.101729.

[43] Wiesner MR, Lowry GV, Alvarez P, Dionysiou D, Biswas P. Assessing the risks of manufactured nanomaterials. Environ Sci Technol. 2006;14:4336–45. 10.1021/es062726m. 10.1021/es062726m 16903268

[44] Shojaei TR, Salleh MA, Tabatabaei M, Mobli H, Aghbashlo M, Rashid SA, Tan T. Applications of nanotechnology and carbon nanoparticles in agriculture. In: Abdul Rashid S, Othman RNIR, Hussein MZ, editors. Synthesis, technology and applications of carbon nanomaterials. Micro and nano technologies. Elsevier B.V.; 2019. pp. 247–277. 10.1016/B978-0-12-815757-2.00011-5.

[45] Yang Z, Yan H, Yang H, Li H, Li A, Cheng R. Flocculation performance and mechanism of graphene oxide for removal of various contaminants from water. Water Res. 2013;47(9):3037–46. 10.1016/j.watres.2013.03.027. 23561497 10.1016/j.watres.2013.03.027

[46] Wang J, Zhang P, Liang B, Liu Y, Xu T, Wang L, Cao B, Pan K. Graphene oxide as an effective barrier on a porous nanofibrous membrane for water treatment. ACS Appl Mater Interfaces. 2016;8(9):6211–8. 10.1021/acsami.5b12723. 26849085 10.1021/acsami.5b12723

[47] Henriques B, Gonçalves G, Emami N, Pereira E, Vila M, Marques PA. Optimized graphene oxide foam with enhanced performance and high selectivity for mercury removal from water. J Hazard Mater. 2016;301:453–61. 10.1016/j.jhazmat.2015.09.028. 26410274 10.1016/j.jhazmat.2015.09.028

[48] Szunerits S, Boukherroub R. Antibacterial activity of graphene-based materials. J Mater Chem B. 2016;4:6892–912. 10.1039/C6TB01647B. 32263558 10.1039/c6tb01647b

[49] Yousefi M, Dadashpour M, Hejazi M, Hasanzadeh M, Behnam B, de la Guardia M, Shadjou N, Mokhtarzadeh A. Anti-bacterial activity of graphene oxide as a new weapon nanomaterial to combat multidrug-resistance bacteria. Mater Sci Eng C. 2017;74:568–81. 10.1016/j.msec.2016.12.125. 10.1016/j.msec.2016.12.125 28254332

[50] Hamk M, Akçay FA, Avcı A. Green synthesis of zinc oxide nanoparticles using *Bacillus subtilis* ZBP4 and their antibacterial potential against foodborne pathogens. Prep Biochem Biotech. 2023;53(3):255–64. 10.1080/10826068.2022.2076243. 10.1080/10826068.2022.2076243 35616319

[51] Hummers WS Jr, Offeman RE. Preparation of graphitic oxide. J Am Chem Soc. 1958;80:1339. 10.1021/ja01539a017.

[52] Rosnan N, Haan TY, Mohammad AW. Synthesis and characterization of ZnO-decorated GO nanocomposite material with different ZnO loading through sol-gel method. J Kejuruter. 2018;30:249–55. 10.17576/jkukm-2018-30(2).

[53] Agnihotri S, Mukherji S, Mukherji S. Antimicrobial chitosan–PVA hydrogel as a nanoreactor and immobilizing matrix for silver nanoparticles. Appl Nanosci. 2012;2:179–88. 10.1007/s13204-012-0080-1.

[54] Dhiman NK, Agnihotri S. Hierarchically aligned nano silver/chitosan–PVA hydrogel for point-of-use water disinfection: contact-active mechanism revealed. Environ Sci Nano. 2020;7:2337–50. 10.1039/D0EN00405G.

[55] CLSI (Clinical and Laboratory Standards Institute). Performance standards for antimicrobial susceptibility testing. In: Performance standards for antimicrobial susceptibility testing: approved standard, 27th Edition. Clinical and Laboratory Standards Institute, Wayne, Pennsylvania, USA. 2017.

[56] CLSI document M2-A9. Performance standards for antimicrobial disk susceptibility tests: Approved standard 9th Edition, Clinical and Laboratory Standards Institute, Wayne, Pennsylvania, USA. 2006.

[57] CLSI. Methods for dilution antimicrobial susceptibility test for bacteria that grow aerobically. Wayne, Pennsylvania, USA: Clinical and Laboratory Standards Institute; 2000.

[58] Mosmann T. Rapid colorimetric assay for cellular growth and survival: application to proliferation and cytotoxicity assays. J Immunol Methods. 1983;65:55–63. 10.1016/0022-1759(83)90303-4. 6606682 10.1016/0022-1759(83)90303-4

[59] WHO. Guidelines for drinking water quality [Electronic Resource]: Incorporating the first and second addenda., 4th ed, WHO. Geneva, Switzerland; 2022. Available online: https://www.who.int/publications/i/item/9789240045064

[60] APHA (American Public Health Association). Standard methods for the examination of water and wastewater. 23rd Edition. Bridgewater LL, Baird RB, Eaton AD, Rice EW, APHA, American Water Works Association., Water Environment Federation, Eds. Washington, DC, USA; 2017. ISBN 978-0-87553-287-5.

[61] O’connor BP. SPSS and SAS programs for determining the number of components using parallel analysis and Velicer’s MAP test. Behav Res Methods Instruments Comput. 2000;32:396–402. 10.3758/BF03200807. 10.3758/bf03200807 11029811

[62] Zhang R, Carlsson F, Edman M, Hummelgård M, Jonsson BG, Bylund D, Olin H. *Escherichia coli* bacteria develop adaptive resistance to antibacterial ZnO nanoparticles. Adv Biosyst. 2018;2:1800019. 10.1002/adbi.201800019. 10.1002/adbi.201800019 33103858

[63] Graves JL Jr, Tajkarimi M, Cunningham Q, Campbell A, Nonga H, Harrison SH, Barrick JE. Rapid evolution of silver nanoparticle resistance in *Escherichia coli*. Front Genet. 2015;6:42. 10.3389/fgene.2015.00042. 25741363 10.3389/fgene.2015.00042 PMC4330922

[64] Yang Y, Mathieu JM, Chattopadhyay S, Miller JT, Wu T, Shibata T, Guo W, Alvarez PJ. Defense mechanisms of *Pseudomonas aeruginosa* PAO1 against quantum dots and their released heavy metals. ACS Nano. 2012;6:6091–8. 10.1021/nn3011619. 22632375 10.1021/nn3011619

[65] Niño-Martínez N, Salas Orozco MF, Martínez-Castañón GA, Torres Méndez F, Ruiz F. Molecular mechanisms of bacterial resistance to metal and metal oxide nanoparticles. Int J Mol Sci. 2019;20:2808. 10.3390/ijms20112808. 31181755 10.3390/ijms20112808 PMC6600416

[66] Balderrama-González AS, Piñón-Castillo HA, Ramírez-Valdespino CA, Landeros-Martínez LL, Orrantia-Borunda E, Esparza-Ponce HE. Antimicrobial resistance and inorganic nanoparticles. Int J Mol Sci. 2021;22:12890. 10.3390/ijms222312890. 34884695 10.3390/ijms222312890 PMC8657868

[67] Rakhshaei R, Namazi H, Hamishehkar H, Kafil HS, Salehi R. In situ synthesized chitosan–gelatin/ZnO nanocomposite scaffold with drug delivery properties: higher antibacterial and lower cytotoxicity effects. J Appl Polym Sci. 2019;136:47590. 10.1002/app.47590.

[68] Ali AA, Asif MA, Mashrai AM, Khanam HK. Green synthesis of ZnO nanoparticles using *Bacillus subtilis* and their catalytic performance in the one-pot synthesis of steroidal thiophenes. Euro Chem Bull. 2014;3:939–45. https://www.eurchembull.com/uploads/paper/393debd32c4c72f0330db83a938ee1d5.pdf.

[69] Hussein MZ, Azmin WH, Mustafa M, Yahaya AH. *Bacillus cereus* as a biotemplating agent for the synthesis of zinc oxide with raspberry-and plate-like structures. J Inorg Biochem. 2009;103:1145–50. 10.1016/j.jinorgbio.2009.05.016. 19577306 10.1016/j.jinorgbio.2009.05.016

[70] Barani M, Masoudi M, Mashreghi M, Makhdoumi A, Eshghi H. Cell-free extract assisted synthesis of ZnO nanoparticles using aquatic bacterial strains: Biological activities and toxicological evaluation. Int J Pharm. 2021;606:120878. 10.1016/j.ijpharm.2021.120878. 34265392 10.1016/j.ijpharm.2021.120878

[71] Hameed H, Waheed A, Sharif MS, Saleem M, Afreen A, Tariq M, Kamal A, Al-Onazi WA, Al Farraj DA, Ahmad S, Mahmoud RM. Green synthesis of zinc oxide (ZnO) nanoparticles from green algae and their assessment in various biological applications. Micromachines. 2023;14(5):928. 10.3390/mi14050928. 37241552 10.3390/mi14050928 PMC10224014

[72] Hu T, Chen L, Yuan K, Chen Y. Poly (N-vinylpyrrolidone)‐decorated reduced graphene oxide with ZnO grown *in situ* as a cathode buffer layer for polymer solar cells. Chem Eur J. 2014;20:17178–84. 10.1002/chem.201404025. 25345881 10.1002/chem.201404025

[73] Zhang L, Li X, Chen S, Guan J, Guo Y, Yu W. 3D chitosan/GO/ZnO hydrogel with enhanced photocorrosion-resistance and adsorption for efficient removal of typical water-soluble pollutants. Catal Commun. 2023;176:106627. 10.1016/j.catcom.2023.106627.

[74] Durmus Z, Kurt BZ, Durmus A. Synthesis and characterization of graphene oxide/zinc oxide (GO/ZnO) nanocomposite and its utilization for photocatalytic degradation of basic fuchsin dye. ChemistrySelect. 2019;4(1):271–8. 10.1002/slct.201803635.

[75] Jain D, Shivani, Bhojiya AA, Singh H, Daima HK, Singh M, Mohanty SR, Stephen BJ, Singh A. Microbial fabrication of zinc oxide nanoparticles and evaluation of their antimicrobial and photocatalytic properties. Front Chem. 2020;8:778. 10.3389/fchem.2020.00778. 33195020 10.3389/fchem.2020.00778 PMC7554571

[76] Bhattacharjee S. DLS and zeta potential–what they are and what they are not? J Control Release. 2016;235:337–51. 10.1016/j.jconrel.2016.06.017. 27297779 10.1016/j.jconrel.2016.06.017

[77] Kim KM, Choi MH, Lee JK, Jeong J, Kim YR, Kim MK, Paek SM, Oh JM. Physicochemical properties of surface charge-modified ZnO nanoparticles with different particle sizes. Int J Nanomed. 2014;9:41–56. 10.2147/IJN.S57923. 10.2147/IJN.S57923 PMC4279853 25565825

[78] El-Nour AT, Abou-Dobara MI, El-Sayed AK, El-Zahed MM. Extracellular biosynthesis and antimicrobial activity of *Bacillus subtilis* ATCC 6633 zinc oxide nanoparticles. SJDFS. 2023;12:39–47. 10.21608/sjdfs.2023.173178.1064.

[79] Huang ZH, Liu G, Kang F. Glucose-promoted Zn-based metal–organic framework/graphene oxide composites for hydrogen sulfide removal. ACS Appl Mater Interfaces. 2012;4:4942–7. 10.1021/am3013104. 22948163 10.1021/am3013104

[80] Khayatian SA, Kompany A, Shahtahmassebi N, Khorsand Zak A. Enhanced photocatalytic performance of Al-doped ZnO NPs-reduced graphene oxide nanocomposite for removing of methyl orange dye from water under visible-light irradiation. J Inorg Organomet Polym Mater. 2018;28:2677–88. 10.1007/s10904-018-0940-6.

[81] Jayachandiran J, Raja A, Arivanandhan M, Jayavel R, Nedumaran D. A facile synthesis of hybrid nanocomposites of reduced graphene oxide/ZnO and its surface modification characteristics for ozone sensing. J Mater Sci Mater Electron. 2018;29:3074–86. 10.1007/s10854-017-8239-x.

[82] Mahdi ZS, Talebnia Rowshan F, Nikzad M, Zamani S. Biosynthesis of zinc oxide nano-rods using *Xanthomonas campestris*. J Mol Biol. 2017;6:15–25. https://doaj.org/article/666bd7b1d5884c3383720f2e89dd7047

[83] Saleh F, Kheirandish F, Hosseini F, Yazdian F. Evaluation of the effect of ZnO nanoparticle derived *Bacillus subtilis* on the expression of efflux pump genes (AdeB AdeRS) in *Acinetobacter baumannii*. J Environ Health sci. 2021;19:1133–41. 10.1007/s40201-021-00679-w. 10.1007/s40201-021-00679-w PMC8172699 34150300

[84] Lozinsky VI, Galaev IY, Plieva FM, Savina IN, Jungvid H, Mattiasson B. Polymeric cryogels as promising materials of biotechnological interest. Trends Biotechnol. 2003;21:445–51. 10.1016/j.tibtech.2003.08.002. 14512231 10.1016/j.tibtech.2003.08.002

[85] Sihombing YA, Nafisah N, Anshori I, Hapidin DA, Edikresnha D, Khairurrijal K. Preparation and characterization of PVA/chitosan-based hydrogels enriched with carbon materials via the freeze-thaw method. In: J Phys: Conference Series 2024 (Vol. 2733, No. 1, p. 012011). IOP Publishing.

[86] Vicentini DS, Smania A Jr, Laranjeira MC. Chitosan/poly (vinyl alcohol) films containing ZnO nanoparticles and plasticizers. Mater Sci Eng C. 2010;30:503–8. 10.1016/j.msec.2009.01.026.

[87] Surkatti R, van Loosdrecht MC, Hussein IA, El-Naas MH. PVA-TiO_2_ nanocomposite hydrogel as immobilization carrier for gas-to-liquid wastewater treatment. Nanomaterials. 2024;14:249. 10.3390/nano14030249. 38334520 10.3390/nano14030249 PMC10856303

[88] Khodaee Z, Mazinani S, Sharif F. Reduced graphene oxide-modified polyvinyl alcohol hydrogel with potential application as skin wound dressings. J Polym Res. 2023;30:5. 10.1007/s10965-022-03384-w.

[89] Lemire JA, Harrison JJ, Turner RJ. Antimicrobial activity of metals: mechanisms, molecular targets and applications. Nat Rev Microbiol. 2013;11:371–84. 10.1038/nrmicro3028. 23669886 10.1038/nrmicro3028

[90] Bhaisare ML, Wu BS, Wu MC, Khan MS, Tseng MH, Wu HF. MALDI MS analysis, disk diffusion and optical density measurements for the antimicrobial effect of zinc oxide nanorods integrated in graphene oxide nanostructures. Biomater Sci. 2016;4:183–94. 10.1039/C5BM00342C. 26575840 10.1039/c5bm00342c

[91] Elbasuney S, El-Sayyad GS, Tantawy H, Hashem AH. Promising antimicrobial and antibiofilm activities of reduced graphene oxide-metal oxide (RGO-NiO, RGO-AgO, and RGO-ZnO) nanocomposites. RSC Adv. 2021;11:25961–75. 10.1039/D1RA04542C. 35479482 10.1039/d1ra04542c PMC9037130

[92] Krishnamoorthy K, Veerapandian M, Zhang LH, Yun K, Kim SJ. Antibacterial efficiency of graphene nanosheets against pathogenic bacteria via lipid peroxidation. J Phys Chem C. 2012;116:17280–7. 10.1021/jp3047054.

[93] Chowdhuri AR, Tripathy S, Chandra S, Roy S, Sahu SK. A ZnO decorated chitosan–graphene oxide nanocomposite shows significantly enhanced antimicrobial activity with ROS generation. RSC Adv. 2015;5:49420–8. 10.1039/C5RA05393E.

[94] Hosseinkhani O, Hamzehlouy A, Dan S, Sanchouli N, Tavakkoli M, Hashemipour H. Graphene oxide/ZnO nanocomposites for efficient removal of heavy metal and organic contaminants from water. Arab J Chem. 2023;16:105176. 10.1016/j.arabjc.2023.105176.

[95] El-Fallal AA, Elfayoumy RA, El-Zahed MM. Antibacterial activity of biosynthesized zinc oxide nanoparticles using Kombucha extract. SN Appl Sci. 2023;5:332. 10.1007/s42452-023-05546-x.

[96] Rajeswari R, Prabu HG. Synthesis characterization, antimicrobial, antioxidant, and cytotoxic activities of ZnO nanorods on reduced graphene oxide. J Inorg Organomet Polym Mater. 2018;28:679–93. 10.1007/s10904-017-0711-9.

[97] Qi YY, Tai ZX, Sun DF, Chen JT, Ma HB, Yan XB, Liu B, Xue QJ. Fabrication and characterization of poly (vinyl alcohol)/graphene oxide nanofibrous biocomposite scaffolds. J Appl Polym Sci. 2013;127:1885–94. 10.1002/app.37924.

[98] Prema D, Prakash J, Vignesh S, Veluchamy P, Ramachandran C, Samal DB, Oh DH, Sahabudeen S, Devanand Venkatasubbu G. Mechanism of inhibition of graphene oxide/zinc oxide nanocomposite against wound infection causing pathogens. Appl Nanosci. 2020;10:827–49. 10.1007/s13204-019-01152-9.

[99] Brandão NL, Portela MB, Maia LC, Antônio A, Silva VL, Silva EM. Model resin composites incorporating ZnO-NP: activity against S. mutans and physicochemical properties characterization. J Appl Oral Sci. 2018;26:e20170270. 10.1590/1678-7757-2017-0270. 29742262 10.1590/1678-7757-2017-0270 PMC5933836

[100] Esmailzadeh H, Sangpour P, Shahraz F, Hejazi J, Khaksar R. Effect of nanocomposite packaging containing ZnO on growth of *Bacillus subtilis* and *Enterobacter aerogenes*. Mater Sci Eng C. 2016;58:1058–63. 10.1016/j.msec.2015.09.078. 10.1016/j.msec.2015.09.078 26478403

[101] Stanković A, Dimitrijević S, Uskoković D. Influence of size scale and morphology on antibacterial properties of ZnO powders hydrothemally synthesized using different surface stabilizing agents. Colloids Surf B Biointerfaces. 2013;102:21–8. 10.1016/j.colsurfb.2012.07.033. 23010107 10.1016/j.colsurfb.2012.07.033

[102] Nandhini J, Karthikeyan E, Rajeshkumar S. Eco-friendly bio-nanocomposites: pioneering sustainable biomedical advancements in engineering. Discover Nano. 2024;19:86. 10.1186/s11671-024-04007-7. 38724698 10.1186/s11671-024-04007-7 PMC11082105

[103] Zhang R, Wang Y, Ma D, Ahmed S, Qin W, Liu Y. Effects of ultrasonication duration and graphene oxide and nano-zinc oxide contents on the properties of polyvinyl alcohol nanocomposites. Ultrason Sonochem. 2019;59:104731. 10.1016/j.ultsonch.2019.104731. 31442767 10.1016/j.ultsonch.2019.104731

[104] Le HN, Nguyen TB, Nguyen DT, Dao TB, Do Nguyen T, Thuc CN. Sonochemical synthesis of bioinspired graphene oxide–zinc oxide hydrogel for antibacterial painting on biodegradable polylactide film. Nanotechnology. 2024;35:305601. 10.1088/1361-6528/ad40b8. 10.1088/1361-6528/ad40b8 38640906

[105] Abd El-Mohdy HL, Aly HM. Characterization, properties and antimicrobial activity of radiation induced phosphorus-containing PVA hydrogels. Arab J Sci Eng. 2023;48:341–51. 10.1007/s13369-022-07031-w.

[106] Khan MQ, Kharaghani D, Nishat N, Shahzad A, Hussain T, Khatri Z, Zhu C, Kim IS. Preparation and characterization of multifunctional PVA/ZnO nanofibers composite membranes for surgical gown application. J Mater Res Technol 20191;8:1328–34. 10.1016/j.jmrt.2018.08.013

[107] Gutha Y, Pathak JL, Zhang W, Zhang Y, Jiao X. Antibacterial and wound healing properties of chitosan/poly (vinyl alcohol)/zinc oxide beads (CS/PVA/ZnO). Int J Biol Macromol. 2017;103:234–41. 10.1016/j.ijbiomac.2017.05.020. 28499948 10.1016/j.ijbiomac.2017.05.020

[108] Abdeen ZI, El Farargy AF, Negm NA. Nanocomposite framework of chitosan/polyvinyl alcohol/ZnO: Preparation, characterization, swelling and antimicrobial evaluation. J Mol Liq. 2018;250:335–43. 10.1016/j.molliq.2017.12.032.

[109] Liu S, Zeng TH, Hofmann M, Burcombe E, Wei J, Jiang R, Kong J, Chen Y. Antibacterial activity of graphite, graphite oxide, graphene oxide, and reduced graphene oxide: membrane and oxidative stress. ACS Nano. 2011;5:6971–80. 10.1021/nn202451x. 21851105 10.1021/nn202451x

[110] Tu Y, Lv M, Xiu P, Huynh T, Zhang M, Castelli M, Liu Z, Huang Q, Fan C, Fang H, Zhou R. Destructive extraction of phospholipids from *Escherichia coli* membranes by graphene nanosheets. Nat Nanotechnol. 2013;8:594–601. 10.1038/nnano.2013.125. 23832191 10.1038/nnano.2013.125

[111] Gurunathan S, Han JW, Dayem AA, Eppakayala V, Kim JH. Oxidative stress-mediated antibacterial activity of graphene oxide and reduced graphene oxide in *Pseudomonas aeruginosa*. Int J Nanomed. 2012;30:5901–14. 10.2147/IJN.S37397. 10.2147/IJN.S37397 PMC3514835 23226696

[112] Mansur HS, Costa ED Jr, Mansur AA, Barbosa-Stancioli EF. Cytocompatibility evaluation in cell-culture systems of chemically crosslinked chitosan/PVA hydrogels. Mater Sci Eng: C. 2009;29:1574–83. 10.1016/j.msec.2008.12.012.

[113] Hemati H, Haghiralsadat F, Hemati M, Sargazi G, Razi N. Design and evaluation of liposomal sulforaphane-loaded polyvinyl alcohol/polyethylene glycol (PVA/PEG) hydrogels as a novel drug delivery system for wound healing. Gels. 2023;9:748. 10.3390/gels9090748. 37754429 10.3390/gels9090748 PMC10529978

[114] Abdallah OM, EL-Baghdady KZ, Khalil MM, El Borhamy MI, Meligi GA. Antibacterial, antibiofilm and cytotoxic activities of biogenic polyvinyl alcohol-silver and chitosan-silver nanocomposites. J Polym Res. 2020;27:1–9. 10.1007/s10965-020-02050-3.

[115] Abed Shlaka W, Saeed S. Gold and silver nanoparticles with modified chitosan/PVA: synthesis, study the toxicity and anticancer activity. Nanomed Res J. 2023;8:231–45. 10.22034/nmrj.2023.03.002.

[116] Guo Y, Dundas CM, Zhou X, Johnston KP, Yu G. Molecular engineering of hydrogels for rapid water disinfection and sustainable solar vapor generation. Adv Mater. 2021;33:2102994. 10.1002/adma.202102994. 10.1002/adma.202102994 34292641

[117] Chen S, De Guzman MR, Tsou CH, Li M, Suen MC, Gao C, Tsou CY. Hydrophilic and absorption properties of reversible nanocomposite polyvinyl alcohol hydrogels reinforced with graphene-doped zinc oxide nanoplates for enhanced antibacterial activity. Polym J. 2023;55:45–61. 10.1038/s41428-022-00711-2.

[118] Zeng X, McCarthy DT, Deletic A, Zhang X. Silver/reduced graphene oxide hydrogel as novel bactericidal filter for point-of‐use water disinfection. Adv Funct Mater. 2015;25:4344–51. 10.1002/adfm.201501454.

[119] Zhang W, Huang H, Bernstein R. Zwitterionic hydrogel modified reduced graphene oxide/ZnO nanocomposite blended membrane with high antifouling and antibiofouling performances. J Colloid Interface Sci. 2022;613:426–34. 10.1016/j.jcis.2021.12.194. 35042040 10.1016/j.jcis.2021.12.194

[120] EWQS (Egyptian Drinking Water Quality Standards). Egyptian standards for drinking water and domestic uses [Electronic Resource in Arabic Language]: Decree of Health Ministry (No. 458; EWQS: Cairo, Egypt; 2007. Available online: https://faolex.fao.org/docs/pdf/egy83626.pdf

[121] Erdem A, Ngwabebhoh FA, Yildiz U. Novel macroporous cryogels with enhanced adsorption capability for the removal of Cu(II) ions from aqueous phase: modelling, kinetics and recovery studies. J Environ Chem Eng. 2017;5:1269–80. 10.1016/j.jece.2017.02.011.

[122] Baimenov A, Berillo D, Abylgazina L, Poulopoulos SG, Inglezakis VJ. Novel amphoteric cryogels for Cd^2+^ ions removal from aqueous solutions. Key Eng Mater. 2018;775:376–82. 10.4028/www.scientific.net/KEM.775.376.

[123] Jalilzadeh M, Şenel S. Removal of Cu(II) ions from water by ion-imprinted magnetic and non-magnetic cryogels: a comparison of their selective Cu(II) removal performances. J Water Process Eng. 2016;13:143–52. 10.1016/j.jwpe.2016. 08.010.

[124] Kurozumi M, Yano Y, Kiyoyama S, Kumar A, Shiomori K. Adsorption properties of arsenic(V) by polyacrylamide cryogel containing iron hydroxide oxide particles prepared by *in situ* method. Resour Process. 2015;62:17–23. 10.4144/rpsj.62.17.

[125] Savina IN, Otero-Gonzalez L, Berillo D. Macroporous cryogel-based systems for water treatment applications and safety: nanocomposite-based cryogels and bacteria-based bioreactors. In: Mohanan PV, Kappalli S, editors. Biomedical applications and toxicity of nanomaterials. Singapore: Springer Nature Singapore; 2023. pp. 1–49. 10.1007/978-981-19-7834-0_1.

[126] Demir D, Vaseashta A, Bölgen N. Macroporous cryogels for water purification. In: Vaseashta A, Maftei C, editors. Water safety, security and sustainability: threat detection and mitigation. Cham, Switzerland: Springer International Publishing; 2021. pp. 275–90. 10.1007/978-3-030-76008-3_12.

[127] Yeo H, Jung J, Song HJ, Choi YM, Wee JH, You NH, Joh HI, Yang CM, Goh M. Preparation and formation mechanism of porous carbon cryogel. Microporous Mesoporous Mater. 2017;245:138–46. 10.1016/j.micromeso.2017.02.075.

